# Intravesical Disitamab Vedotin (RC48) for HER2‐Expressing High‐Risk Non‐Muscle‐Invasive Bladder Cancer: A Single‐Arm, Dose–Escalation Phase I Trial Study

**DOI:** 10.1002/mco2.70288

**Published:** 2025-07-13

**Authors:** Xu Chen, Ming Huang, Zehua Chen, Chengjunyu Zhang, Bolin Pan, Chu Liu, Wentong Xu, Jianmin Fang, Jiang Huang, Tianxin Lin

**Affiliations:** ^1^ Department of Urology Sun Yat‐sen Memorial Hospital Sun Yat‐sen University Guangzhou China; ^2^ Guangdong Provincial Key Laboratory of Malignant Tumor Epigenetics and Gene Regulation Sun Yat‐Sen Memorial Hospital Sun Yat‐Sen University Guangzhou China; ^3^ Guangdong Provincial Clinical Research Center for Urological Diseases Guangzhou China; ^4^ RemeGen, Ltd. Yantai Shandong China; ^5^ School of Life Science and Technology Tongji University Shanghai China

**Keywords:** antibody‒drug conjugates (ADC), Disitamab vedotin (RC48), efficacy, intravesical, non‐muscle‐invasive bladder cancer, safety

## Abstract

HER2 expression is correlated with diminished efficacy of Bacillus Calmette–Guérin (BCG) instillation in high‐risk non‐muscle‐invasive bladder cancer (HR‐NMIBC). The development of effective intravesical treatments for HER2‐expressing HR‐NMIBC is of great urgency. In this single‐arm phase I trial (ChiCTR2300073975), HER2‐expressing HR‐NMIBC patients received an induction course of weekly intravesical Disitamab vedotin (RC48) following a 3+3 design (60, 120, or 180 mg) for 6 weeks, followed by optional maintenance dose monthly for 11 sessions. The primary objective was to assess the safety and tolerability of intravesical RC48. The secondary objective was to determine the oncological outcomes. Between August 2023 and March 2024, nine patients were enrolled, and all completed the induction course without dose‐limiting toxicities (DLTs) or grade ≥3 drug‐related adverse events (AEs). The reported drug‐related AEs included urinary tract infection (55.6%, 5/9), urinary frequency (11.1%, 1/9), and hematuria (11.1%, 1/9). The 6‐month and 12‐month recurrence‐free survival (RFS) rates were 100% (8/8) and 87.5% (7/8), respectively, whereas the progression‐free survival (PFS) rates were 100% (8/8) and 100% (8/8). Taken together, these findings indicate that intravesical RC48 was well tolerated and showed preliminary efficacy in HER2‐expressing HR‐NMIBC. The maximum tolerated dose was not reached, and further dose exploration is ongoing (NCT06378242).

## Graphical abstract

Intravesical RC48 is well tolerated, with no serious drug‐related AEs or DLTs, and exhibits effective anti‐tumor activity in HER2‐expressing HR‐NMIBC patients.

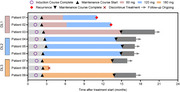

## 1 Introduction

Bladder cancer (BCa) is the second most prevalent urinary tract carcinoma, with approximately 549,000 new cases diagnosed globally in 2021 [1, 2]. Non‐muscle invasive bladder cancer (NMIBC) makes up 75%–80% of BCa cases and is notable for a high incidence and recurrence rate, especially high‐risk NMIBC (HR‐NMIBC) [3]. For HR‐NMIBC, the recommended treatment involves transurethral resection of the bladder tumor (TURBT) and adjuvant intravesical Bacillus Calmette–Guérin (BCG) instillations. However, the intolerable adverse effects (AEs), as well as the internationally recognized shortage of BCG, limit the widespread use of intravesical BCG in HR‐NMIBC [4].

Recent studies revealed that approximately 70%–85% of NMIBC patients are HER2 positive [5, 6], and approximately 10% exhibit HER2 alterations (including driver mutations or amplifications) [7]. Notably, HER2 expression was associated with reduced recurrence‐free survival (RFS) and progression‐free survival (PFS) in patients with NMIBC [8]. High HER2 expression was correlated with diminished efficacy of BCG treatments [5]. However, HER2‐targeting therapies, including monoclonal antibodies and tyrosine kinase inhibitors (TKIs), have not demonstrated significant clinical benefits in urothelial carcinoma (UC) [9]. Although the FDA has approved chemotherapy (e.g., gemcitabine or epirubicin) and immunotherapy (pembrolizumab) [10], several intravesical therapies, including TAR‐200 (a gemcitabine delivery system) and CG0070 (an adenovirus targeting BCa cells), have received FDA breakthrough therapy designation in the treatment of HR‐NMIBC [11, 12], intravesical treatment for HER2‐expressing HR‐NMIBC remains a challenge, necessitating innovative strategies to improve patient outcomes.

Disitamab vedotin (RC48), an anti‐HER2 antibody–drug conjugate (ADC), has been approved in China for the treatment of platinum‐refractory metastatic UC (mUC) [13]. RC48 exhibits multiple anti‐tumor mechanisms. The antibody component of RC48 binds to HER2 on the tumor cell surface, triggering endocytosis and the release of monomethyl auristatin E (MMAE), which disrupts the intracellular microtubule network, leading to mitotic cell cycle arrest and apoptosis. Additionally, the released MMAE diffuses into the tumor microenvironment, inducing bystander cytotoxicity by targeting adjacent dividing cells, even those with low or heterogeneous HER2 expression [14]. Our previous study demonstrated that intravesical RC48 had superior anti‐tumor effects compared with disitamab or epirubicin in an orthotopic BCa mouse model with manageable toxicity [6]. These results indicate that, compared with HER2 monoclonal antibodies or TKIs, RC48 has superior tumor‐killing efficacy in bladder cancer. However, the clinical safety and efficacy of intravesical RC48 in HR‐NMIBC remain unknown.

In this study, we initiated an open‐label, single‐arm, dose–escalation phase I study of RC48 in HER2‐expressing HR‐NMIBC patients, which is, to our knowledge, the first study reporting the intravesical treatment of HR‐NMIBC with ADC.

## 2 Results

### 2.1 Baseline Patient Characteristics

**FIGURE 1:**
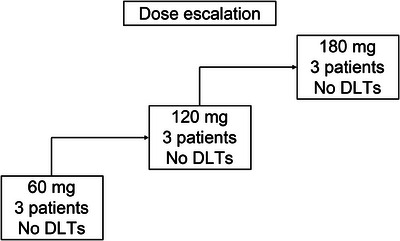
Dose escalation of intravesical Disitamab vedotin (RC48). DLT, dose‐limiting toxicity.

From August 2023 to March 2024, nine patients were enrolled and received the study drug following a “3+3” design at Sun Yat‐sen Memorial Hospital, Guangdong, China (Figure 1). The representativeness of the study participants is summarized in Table S1. The median age of the participants was 69 years (range: 35–72 years). One patient (11.1%) was BCG‐unresponsive, whereas the other eight patients (88.9%) were BCG‐untreated. With respect to HER2 expression levels, three patients (33.3%) had HER2 1+ status, five patients (55.6%) had HER2 2+ status, and one patient (11.1%) had HER2 3+ status. Patient characteristics are detailed in Table 1. Patient 08 discontinued treatment for personal reasons. All nine patients were followed up until April 30, 2025, with a median follow‐up time of 16.97 months (interquartile range [IQR]: 10.97–17.3).

**TABLE 1:** Baseline characteristics of patients.

	All (*n* = 9)	DL1 (*n* = 3)	DL2 (*n* = 3)	DL3 (*n* = 3)
Median age (range)	69 (35–72)	70 (57–72)	69 (35–71)	69 (59–72)
Sex, *n* (%)				
Male	6 (67)	2 (67)	2 (67)	2 (67)
Female	3 (33)	1 (33)	1 (33)	1 (33)
ECOG performance status, *n* (%)				
0	9 (100)	3 (100)	3 (100)	3 (100)
1	0 (0)	0 (0)	0 (0)	0 (0)
Bladder T stages, *n* (%)				
CIS	0 (0)	0 (0)	0 (0)	0 (0)
HG Ta + CIS	0 (0)	0 (0)	0 (0)	0 (0)
HG T1 + CIS	0 (0)	0 (0)	0 (0)	0 (0)
HG Ta	0 (0)	0 (0)	0 (0)	0 (0)
HG T1	9 (100)	3 (100)	3 (100)	3 (100)
Prior BCG treatment, *n* (%)				
BCG unresponsive	1 (11)	1 (33)	0 (0)	0 (0)
BCG untreated	8 (89)	2 (67)	3 (100)	3 (100)
HER2 status, *n* (%)				
HER2 1+	3 (33)	2 (67)	1 (33)	0 (0)
HER2 2+	5 (56)	1 (33)	2 (67)	2 (67)
HER2 3+	1 (11)	0 (0)	0 (0)	1 (33)

Abbreviations: BCG, Bacillus Calmette–Guérin; DL, dose level; ECOG, Eastern Cooperative Oncology Group.

### 2.2 Dose‐Limiting Toxicity (DLT), Maximum Tolerated Dose (MTD), and Recommended Phase 2 Dose (RP2D)

According to the trial protocol (Supporting Information Appendix), the maximum recommended human dose was 180 mg (3.6 mg/mL) or 240 mg (4.8 mg/mL). Considering the safety of the patients, the three dose groups in this study were set as 60, 120, or 180 mg of intravesical RC48. With three patients in each group completing the induction course, no DLTs were observed (Figure 1). The MTD was not reached, and the RP2D could not be determined. Further dose exploration is ongoing (NCT06378242) to determine the MTD and PR2D.

### 2.3 Safety

**FIGURE 2:**
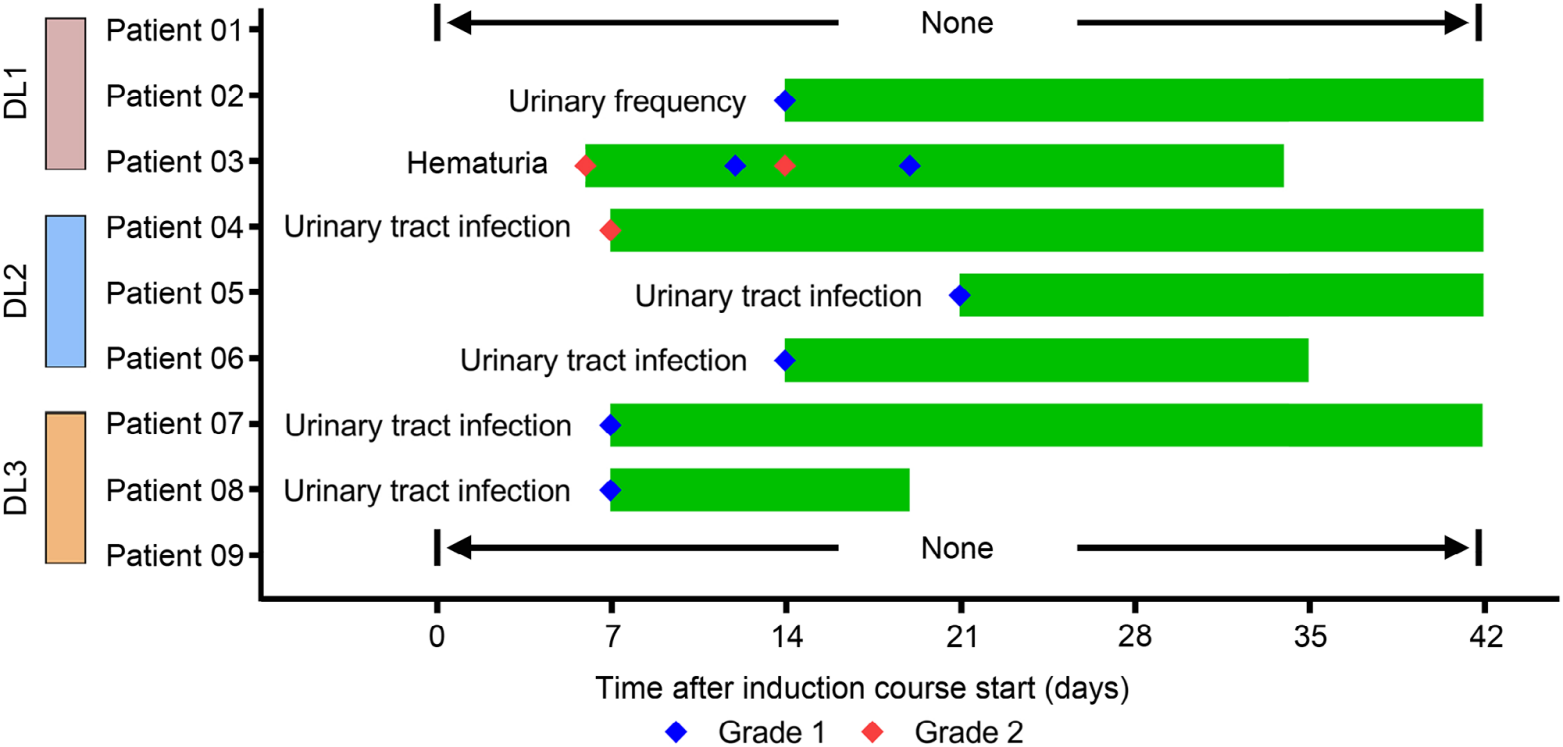
Drug‐related adverse events (AEs) of patients. Each bar represents the AEs of an individual patient as designated. All AEs were diagnosed on the basis of the examination results during follow‐up.

At the cut‐off date of April 30, 2025, seven of the nine patients (77.8%) experienced drug‐related AEs (DRAEs) of any grade. Hematuria in Patient 03 and urinary tract infections (UTIs) in Patients 06 and 08 were resolved during the induction course. However, the urinary frequency in Patient 02 and the UTIs of Patients 04, 05, and 07 persisted through the end of the induction course (Figure 2). Notably, no grade ≥3 AEs were observed (0%, 0/9). The most common DRAE was UTIs, affecting five patients (55.6%), while one patient (11.1%) experienced urinary frequency, and another (11.1%) experienced hematuria (Table 2).

**TABLE 2:** Drug‐related AEs.

Drug related AEs, *n* (%)	All (*n* = 9)	DL1 (*n* = 3)	DL2 (*n* = 3)	DL3 (*n* = 3)
Any grade ≥3 AEs, *n* (%)	0 (0)	0 (0)	0 (0)	0 (0)
Urinary tract infection	5 (55.6)	0 (0)	3 (100)	2 (66.7)
Urinary frequency	1 (11.1)	1 (33.3)	0 (0)	0 (0)
Hematuria	1 (11.1)	1 (33.3)	0 (0)	0 (0)
No AE	2 (22.2)	1 (33.3)	0 (0)	1 (33.3)

*Note*: Data are number (%). All AEs were graded according to NCI CTCAE 5.0.

Abbreviations: AE, adverse events; CTCAE, Common Terminology Criteria for Adverse Events; DL, dose level.

### 2.4 Efficacy

**FIGURE 3:**
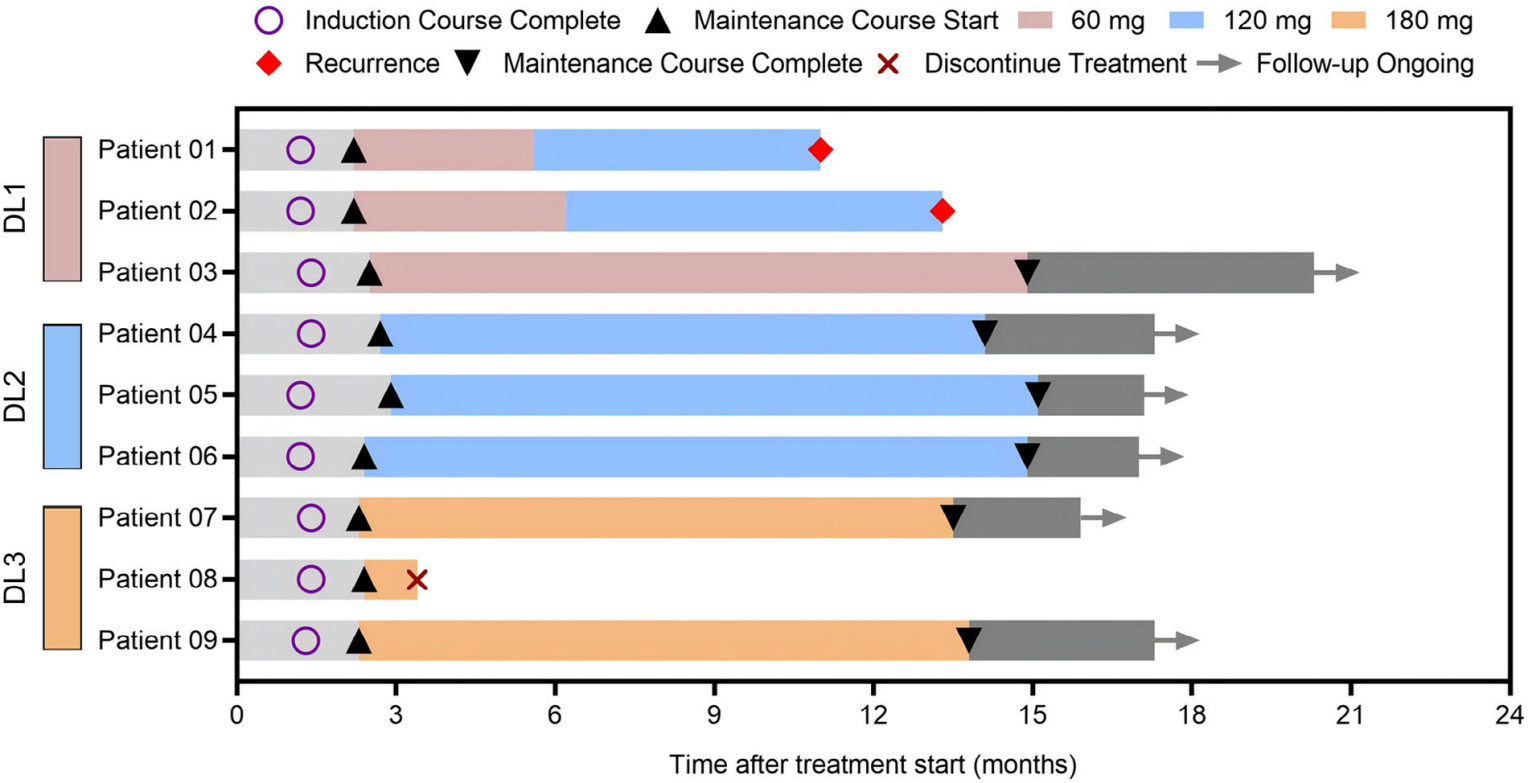
Study Swimmer's plot. Each bar represents an individual subject as designated. DL, dose level.

**FIGURE 4:**
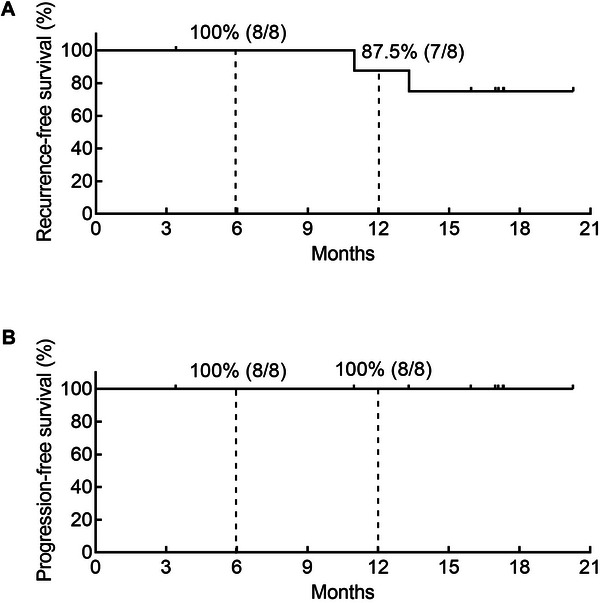
Patient survival. Recurrence‐free survival (A) and Progression‐free survival (B) in the full analysis set.

The preliminary efficacy of intravesical RC48 treatment is summarized in Figure 3. Patient 08 discontinued treatment for personal reasons. As of April 30, 2025, two patients had developed tumor recurrence, while no patients experienced tumor progression. The 6‐month and 12‐month RFS rates were 100% (8/8) and 87.5% (7/8), respectively, with PFS rates of 100% at both time points (8/8 and 8/8, respectively) (Figure 4). Notably, at doses of 120 mg and 180 mg, all participants achieved disease control without experiencing any DLTs.

**FIGURE 5:**
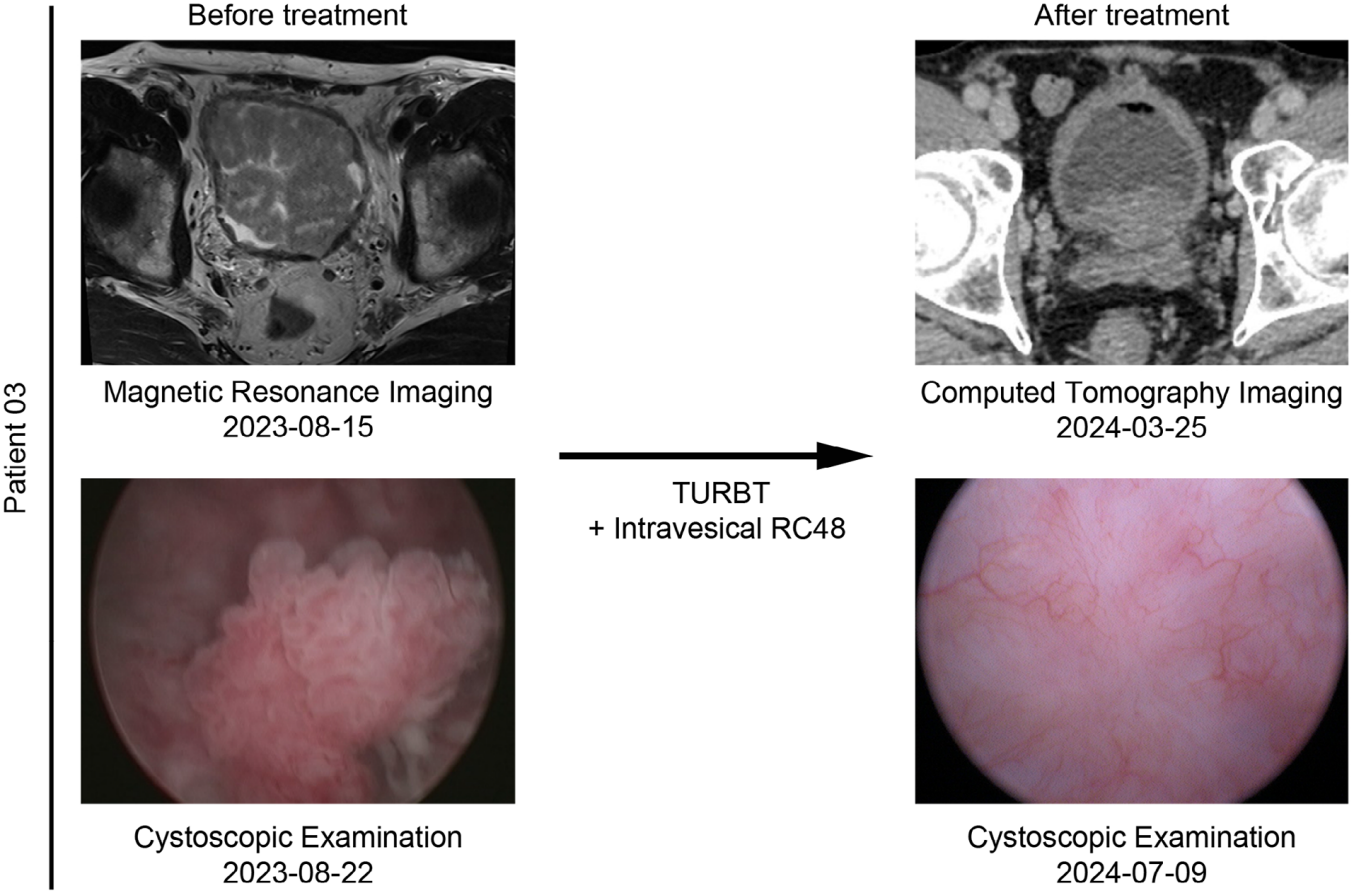
Radiographic imaging and cystoscopic examination of Patient 03. The left panel shows the tumors in the bladder before treatment. The right panel shows the status of the bladder after transurethral resection of the bladder tumor (TURBT) and intravesical RC48 treatment.

Both patients who developed tumor recurrence were in the 60 mg group. Patient 01, who was BCG‐unresponsive, experienced tumor recurrence twice, maintaining a tumor‐free state for 3 months and 7 months. In August 2023, he underwent TURBT and was pathologically diagnosed with stage T1 disease. After completing the induction course and an 8‐month maintenance course of intravesical RC48, the patient underwent TURBT and was confirmed to have stage Ta tumor recurrence in July 2024, maintaining a tumor‐free state for 11 months thereafter. Patient 02, who was newly diagnosed with bladder cancer, was included in this study after TURBT. She completed the induction course and an 11‐month maintenance course, maintaining a tumor‐free state for 13.3 months (Figure 3). Interestingly, Patient 03 demonstrated a good therapeutic response. He had undergone two TURBTs without BCG treatment between August 2018 and January 2019. An ultrasound examination revealed that multiple occupations filled the bladder, with the largest measuring 52 × 40 mm, and a biopsy confirmed recurrence in August 2023. Following a 6‐week induction course and an 11‐month maintenance course of 60 mg, the patient achieved complete remission, as confirmed by ultrasound and cystoscopic examination (Figure 5).

### 2.5 Urine DNA Methylation Assay

Urine DNA methylation assays were conducted to assess residual or recurrent tumors. Eight patients, excluding Patient 08, underwent the assays after completing their induction therapy. The results revealed that Patients 01, 02, 05, 06, and 09 tested positive for methylation, whereas Patients 03, 04, and 07 tested negative. Notably, Patients 01 and 02 experienced tumor recurrence, whereas Patients 05, 06, and 09 were found to have biopsy‐confirmed chronic inflammation. In contrast, Patients 03, 04, and 07 presented no signs of tumor recurrence. Moreover, despite variations in the timing of the assays, the methylation results remained consistent for each patient (Table 3).

**TABLE 3:** Urine DNA methylation assay.

Patient	First assay	Day	Second assay	Day
01	Positive	0	Positive	222
02	Positive	11	Positive	349
03	Negative	160	Negative	285
04	Negative	0	Negative	195
05	Positive	5	–	–
06	Positive	0	–	–
07	Negative	184	–	–
08	–	–	–	–
09	Positive	158	–	–

*Note*: The days were counted since the introduction course finished.

## 3 Discussion

The recommended treatment for HR‐NMIBC consists of TURBT followed by adjuvant intravesical BCG instillations. However, only 50% of patients benefit from this therapy. Previous studies have reported that high HER2 expression is associated with diminished efficacy of BCG treatments [5], and more than 70% of NMIBC patients are HER2 positive [6]. Therefore, significant efforts have been made to identify patients who do not benefit from BCG treatment and to explore alternative therapies to radical surgery [15]. RC48, a targeted ADC for HER2‐positive tumors, has shown promising efficacy in the treatment of mUC. In a combined analysis of the RC48‐C005 and C009 studies, 107 HER2‐positive patients with locally advanced or mUC who had experienced first‐line systemic chemotherapy failure were treated with RC48, achieving an ORR of 50.5%, a median PFS of 5.9 months, and a median OS of 14.2 months [13]. Moreover, RC48‐C017 is a study of RC48 combined with toripalimab for HER2‐low/positive MIBC patients, with a pCR rate of 63.6%, a 12‐month EFS rate of 92.5%, and an OS rate of 95.5% [16]. Given the promising efficacy of RC48 in UC and BCa, RC48 is being investigated for use in neoadjuvant therapy for MIBC. However, its toxicity and efficacy when delivered intravesically for NMIBC require further investigation. This study represents the first clinical trial assessing the feasibility of intravesical RC48 in HER2‐expressing HR‐NMIBC, which showed no serious DRAEs and encouraging efficacy.

In this trial, DRAEs included UTIs (55.6%, 5/9), urinary frequency (11.1%, 1/9), and hematuria (11.1%, 1/9), with no Grade ≥3 AEs observed. These AEs are common complications associated with bladder instillation and are attributed mainly to inflammation of the bladder mucosa following treatment. All AEs in this study were manageable with anti‐infective treatments and either resolved spontaneously or with treatment. The duration of these AEs was generally short and controllable through appropriate management.

The most common DRAEs induced by intravenous RC48 in mUC patients included leukopenia, liver function injury, and peripheral sensory neuropathy. These AEs were attributed mainly to the toxicity of MMAE and were clinically manageable in the current combined analysis [13, 17]. Recent studies suggest that intravesical drug delivery reduces systemic AEs in BCa, with RC48 showing a safety profile similar to that of gemcitabine [18, 19, 20]. In the NCT02808143 trial, BCG‐related AEs were mostly Grade 3, with the most common being hematuria (55.6%) and UTIs (22.2%). Pembrolizumab caused hematuria (44.4%, Grade 1 or 2), although one patient died from treatment‐related myasthenia gravis [21]. In this trial, intravesical RC48 led to a higher rate of UTIs, which was diagnosed by increased white blood cell (WBC) counts in the urine but a lower rate of hematuria. Considering that the WBC count was elevated due to post‐TURBT wound healing, as well as RC48‐induced tumor cell killing, UTI patients without significant urinary irritation symptoms might recover spontaneously. If irritation symptoms occur, they can be alleviated with short‐term antibiotic use. Moreover, for patients with a high urinary frequency, mirabegron was used, while hematuria patients could recover by temporarily stopping instillation and taking hemostatic drugs. Therefore, during the induction course, regular urinalysis is essential. If symptoms or increased WBC counts emerge, patients can recover spontaneously or with medication without ending RC48 illustration.

Current bladder preservation strategies for HR‐NMIBC include intravesical BCG, immunotherapy, chemotherapy, gene therapy (nadofaragene firadenovec), device‐assisted intravesical treatment, and ongoing clinical trials [4]. For HR‐NMIBC patients, intravesical BCG remains the standard treatment, with a 1‐year RFS rate ranging from 60% to 90% and PFS rates ranging from 80% to 90%. A phase II clinical study investigating intravesical gemcitabine plus docetaxel in BCG‐naïve patients reported a 1‐year RFS rate of 92% [22]. Furthermore, to reduce BCG toxicity and enhance efficacy, several clinical trials have proposed combinations of chemotherapy or immunotherapy with BCG, such as atezolizumab + BCG or tirilizumab + cisplatin + BCG. These treatments achieved a 1‐year RFS rate of 80%–90%, but grade ≥3 AEs have been reported [23]. For BCG‐unresponsive NMIBC patients, previous studies reported 12‐month CR rates of 18% for pembrolizumab and 24% for nadofaragene firadenovec in BCG‐unresponsive NMIBC patients [10, 24]. Meghani et al. conducted a 3+3 phase I trial of pembrolizumab + BCG and reported 67% and 22% 6‐month and 12‐month RFS, respectively [21]. The SunRISe‐1 trial, which used intravesical gemcitabine for BCG‐unresponsive HR‐NMIBC patients, reported 76% and 62% estimated 6‐month and 12‐month CR rates, respectively [25]. In our trial, we observed 100% RFS rates at 6‐month and 87.5% at 12‐month, with 100% PFS rates at both 6‐month and 12‐month. Excluding one BCG‐unresponsive patient, the remaining seven BCG‐naïve patients with a full‐year follow‐up showed no recurrence. Patient 01 experienced relapses after BCG or epirubicin treatment and maintained tumor‐free status for 3 and 7 months, respectively. These findings indicate that patients who are BCG unresponsive may have a lower sensitivity to chemotherapy and immunotherapy and might have more widespread tumors, making treatment more challenging. However, intravesical RC48 sustained a longer tumor‐free status with stage Ta tumor recurrence, which was earlier than the previous stage T1. These data demonstrate the superior antitumor activity of RC48 in BCG‐unresponsive NMIBC patients. Additionally, previous studies have reported clinical trials of intravenous RC48 + intravesical BCG for HR‐NMIBC, reporting a 100% 6‐month EFS rate. This result is consistent with our findings, as two cases of grade ≥3 AEs occurred. On the basis of our results and those of existing clinical trials, we plan to investigate the safety and efficacy of the combination of intravenous ICB with intravesical RC48 in HR‐NMIBC patients, especially BCG‐unresponsive patients.

In UC, the ORRs in RC48‐treated patients with immunohistochemical (IHC) 3+, IHC 2+, and FISH+ and those with IHC 2+ and FISH− were 58.8%, 66.7%, and 40%, respectively. The ORR demonstrated a trend toward better efficacy of RC48 in patients with HER2 amplification than in those without amplification (61.5% vs. 44.8%, *p* = 0.059) [26]. Furthermore, owing to RC48's bystander cytotoxic effect on tumor cells adjacent to HER2‐expressing cells, RC48 also exhibits antitumor activity in HER2‐negative or HER2‐low‐expressing patients. A recent phase II study evaluating the efficacy and safety of RC48 in HER2‐negative and low‐expressing mUC reported that, among 19 enrolled patients (6 HER2‐negative and 13 HER2‐low) from September 2019 to September 2022, the ORR was 31.6%, with a disease control rate of 94.7% (18/19). The median PFS and OS were 5.5 months and 16.4 months, respectively [27]. In summary, in UC, patients with high HER2 expression show greater sensitivity to RC48, but RC48 still has antitumor effects on HER2‐negative or HER2‐low‐expressing patients. Although no data currently exist concerning the efficacy of HER2 expression on RC48 in bladder cancer, we hypothesize that this conclusion remains applicable. We will further analyze the impact of HER2 expression on the efficacy of intravesical RC48 in HR‐NMIBC patients through the larger cohort and longer follow‐up of the NCT06378242 study.

Among patients treated with DL1 (Dose Level 1, 60 mg), Patients 01 and 02 had biopsy‐confirmed recurrence after the induction course followed by the maintenance course. Although no recurrence was observed in Patient 03 by the cut‐off date, the efficacy of intravesical RC48 appeared to be dose dependent, which contrasts with its effects in advanced breast cancer [28]. Given that no DLT was observed at a dose of 180 mg and that the MTD was not reached, the dose for the dose‐expansion trial will be determined on the basis of long‐term clinical outcomes in the future.

In this study, the results of the urine DNA methylation assays were positive for Patients 01, 02, 05, 06, and 09. Among them, Patients 01 and 02 experienced tumor recurrence, whereas Patients 05, 06, and 09 showed no evidence of recurrence. Our previous findings revealed an 84.8% positive predictive value for HR‐NMIBC or MIBC [29], highlighting the importance of regular cystoscopies for Patients 05, 06, and 09.

There are several limitations inherent to this single‐center phase I study, including the small sample size and short follow‐up period, which hinder a thorough evaluation of the long‐term efficacy and safety of the treatment. To address these issues, patients in this study will still be followed after treatment to monitor AEs and anti‐tumor efficacy of intravesical RC48, with results updated in a timely manner. Moreover, we launched an open‐label, single‐arm, multicenter phase I/II clinical trial (NCT06378242). A phase I study involves dose escalation at 120, 180, 240, and 360 mg to assess the incidence or severity of DLTs and AEs, as well as to determine RP2D and MTD. The phase II study will evaluate the 12‐month DFS rate, CR rate, and DOR (duration of response) in HER2‐expressing HR‐NMIBC patients who are BCG naïve or unresponsive at RP2D. The project is progressing well.

In conclusion, this first‐in‐human phase I clinical study demonstrated the safety and anti‐tumor activity of intravesical RC48 in HER2‐positive HR‐NMIBC. Given its encouraging efficacy in treating mUC, RC48 could represent a promising option for neoadjuvant therapy in MIBC patients and bladder preservation strategies in HR‐NMIBC patients. Our data indicate that intravesical RC48 is well tolerated, with no serious DRAEs or DLTs, and that it exhibits effective anti‐tumor activity. Further dose‐expansion studies with longer follow‐up periods are ongoing to confirm the safety and efficacy of RC48.

## 4 Materials and Methods

### 4.1 Study Design and Patients

This study was a single‐arm, open‐label, dose–escalation phase I trial conducted at Sun Yat‐sen University, Guangdong, China. This study aimed to evaluate the safety, tolerability, and anti‐tumor activity of intravesical RC48 to determine the MTD and RP2D for the treatment of HER2‐expressing HR‐NMIBC.

The participants were required to be between 18 and 75 years old, with an Eastern Cooperative Oncology Group (ECOG) score of 0–2. Eligible patients were required to be diagnosed with HR‐NIMBC (including T1, Ta or CIS) and receive TURBT within 3 weeks. Prior to enrollment, all grossly visible lesions were resected via endoscopy, and the pathological staging was confirmed at the study institution. The inclusion criteria required that participants either refuse or be deemed medically ineligible for radical cystectomy. All participants were categorized as BCG‐unresponsive or BCG‐untreated. BCG‐unresponsive patients were defined as those with biopsy‐proven recurrence after receiving at least five induction treatments followed by two maintenance treatments of BCG. BCG‐untreated patients, including those who rejected BCG treatment because of serious AEs or harbored intolerant contraindications for BCG treatment, were also included. Another key eligibility criterion was HER2 positivity, which was defined as an IHC score of 1+, 2+, or 3+. The key exclusion criteria included MIBC, previous treatments within 4 weeks (including chemotherapy, radiotherapy, targeted therapy, or immunotherapy) other than TURBT followed by immediate intravesical treatment, and serious conditions such as a history of acute myocardial infarction and congestive heart failure (NYHA) grade ≥3. The full inclusion and exclusion criteria are listed in the Protocol in Supporting Information Appendix.

### 4.2 Procedures

This dose–escalation study followed a “3+3” design, encompassing three dose‐level cohorts. Patients received an induction regimen of intravesical RC48 administered weekly for six consecutive weeks. The starting dose was 60 mg. If no DLTs were observed in the initial cohort of three patients, dose escalation proceeded to the next level, with a maximum planned dose of 180 mg. In the event that one of the initial three patients experienced DLT, an additional three patients were enrolled at the same dose level for further observation. If none of the other three patients exhibited DLTs, dose escalation continued. However, if one or more patients experienced DLTs in the expanded cohort, dose escalation would be halted, and the study would revert to the previous dose level (for the 60 mg cohort, a reduction to 30 mg). If two or more patients developed DLTs at any dose level, the preceding dose was defined as the MTD.

DLT was defined as any grade 3 hematological toxicity (excluding grade 4 neutropenia) or any grade ≥3 nonhematological toxicity occurring within 28 days of the first treatment. The RP2D for intravesical RC48 was determined on the basis of a comprehensive assessment of safety and preliminary efficacy data collected throughout the study. AEs were graded and monitored according to the Common Terminology Criteria for Adverse Events (CTCAE) version 5.0. Clinical response was evaluated via a combination of ultrasound, cystoscopic examination, a urine DNA methylation assay, and biopsy, with assessments conducted at least every 3 months following the start of the induction course.

If there was no recurrence or disease progression within 28 days postinduction, patients were eligible for a maintenance course of intravesical RC48, which was administered once monthly for up to 11 sessions. The dose level for the maintenance phase could be adjusted on the basis of individual response and tolerance, with the option to escalate the dose if appropriate.

### 4.3 Safety Assessments

AEs refer to all adverse medical events that occur in subjects after receiving the test medication, which can manifest as symptoms, signs, diseases, or laboratory abnormalities but are not necessarily related to the test medication. DRAEs refer to AEs that occur after the start of treatment with the test medication or after the exacerbation of preexisting medical conditions before treatment. Severe AEs (SAEs) are those that meet any of the following criteria: resulting in death, life‐threatening, requiring hospitalization or prolonged hospitalization, resulting in permanent or significant disability or loss of function, or congenital anomalies or birth defects, leading to other significant medical events that may not immediately threaten life, death, or hospitalization but require medical intervention to prevent one of the above situations. The detailed definitions of the safety assessments are listed in the Protocol in the Supporting Appendix.

### 4.4 Outcomes

The primary objectives were to evaluate the safety and tolerability of intravesical RC48 in HR‐NMIBC patients with HER2 expression, observe the possible DLTs, determine the MTD, and identify the RP2D. The secondary objectives were the clinical response of these patients, including the RFS rate, PFS rate, and DOR since the induction course started at 3, 6, 18, and 24 months.

### 4.5 Urine DNA Methylation Assay

The Urifind (AnchorDx, China), a diagnostic assay for BCa based on two methylation markers developed in our previous study [29, 30], was used to evaluate the recurrence or progression of HR‐NMIBC after the intravesical RC48 induction course. In brief, urine samples were collected, centrifuged, washed, and resuspended to obtain cell pellets from the urine. After the DNA was isolated from the cell pellets, quantitative PCR was performed to analyze the methylation status of ONECUT2 and VlM. A positive result indicated a high risk of BCa.

### 4.6 Statistical Analysis

The sample size for this study was based on a 3 + 3 design, and three participants were enrolled in each dose group. Patients’ baseline characteristics were summarized via descriptive statistics. The 6‐month and 12‐month RFS and PFS rates with 95% confidence intervals (CIs) were estimated via the Kaplan‒Meier method. All figures were created using GraphPad Prism and Adobe Photoshop. GraphPad Prism was used for statistical and data analysis.

## Author Contributions

X.C., T.L., and J.F. designed the overall study. X.C., M.H., and Z.C. conducted the clinical trial. Z.C., C.Z., and C.L. collected the clinical data, and M.H., B.P., and W.X. analyzed the clinical data. X.C., T.L., and M.H. wrote the manuscript. T.L., J.H., and J.F. supervised the study. All authors have read and approved the final manuscript. All the authors confirm that they had full access to all the data in the study. All the authors accept responsibility for the decision to submit this manuscript for publication.

## Ethics Statement

The study was approved by the Ethics Committee of Sun Yat‐Sen Memorial Hospital of Sun Yat‐Sen University (SYSKY‐2023‐545‐01). This study was registered with ChiCTR2300073975. All patients provided written informed consent prior to enrollment. Data were prospectively collected at the time of the procedure and during the routine care of the patients.

## Conflicts of Interest

All the authors declare no conflicts of interest.

## Supporting information



Supplementary data‐0508.docx

## Data Availability

The data are available upon request from the corresponding author. Data are not publicly available due to patient privacy concerns.
