# Supplementary material for: Intravesical Disitamab Vedotin (RC48) for HER2‐Expressing High‐Risk Non‐Muscle‐Invasive Bladder Cancer: A Single‐Arm, Dose–Escalation Phase I Trial Study
Source: MedComm (2020). 2025 Jul 13;6(7):e70288. doi: 10.1002/mco2.70288 (PMC12256569; doi:10.1002/mco2.70288)
Supplement: Supplementary file 1 — Supplementary data‐0508.docx [file MCO2-6-e70288-s001.docx]

**Supplementary Appendix**

**Intravesical Disitamab vedotin (RC48) for HER2-expressing high-risk non-muscle-invasive bladder cancer: a single-arm, dose-escalation phase I trial study**

Contents

[Results 4](#_Toc197603942)

[Supplementary Table S1. Representativeness of Study Participants. 4](#_Toc197603943)

[Protocol 5](#_Toc197603944)

[Abstract 6](#_Toc197603945)

[1. Background 11](#_Toc197603946)

[1.1 Introduction 11](#_Toc197603947)

[1.2 Bladder Cancer and HER2 15](#_Toc197603948)

[1.3 RC48 (Disitamab vedotin) 16](#_Toc197603949)

[2. Study Purpose and Endpoints 22](#_Toc197603950)

[2.1 Study Purpose 22](#_Toc197603951)

[2.2 Study Endpoints 22](#_Toc197603952)

[3. Study Design 22](#_Toc197603953)

[3.1 Overall Summary 22](#_Toc197603954)

[3.2 Dose-Limiting Toxicity (DLT) 24](#_Toc197603955)

[3.3 Maximum Tolerated Dose (MTD) 25](#_Toc197603956)

[3.4 Dose Escalation Starting Dose and Expected Maximum Dose Determination Basis 25](#_Toc197603957)

[3.5 Dosing Scheme Design Basis 26](#_Toc197603958)

[4. Study participants 26](#_Toc197603959)

[4.1 Inclusion criteria 27](#_Toc197603960)

[4.2 Exclusion criteria 28](#_Toc197603961)

[4.3 Treatment Termination/Early Termination 30](#_Toc197603962)

[4.4 Study Termination/Cessation 31](#_Toc197603963)

[4.5 Subject Loss to Follow-up 31](#_Toc197603964)

[4.6 Subject Numbering and Name Abbreviation 32](#_Toc197603965)

[4.7 Screening Failure 32](#_Toc197603966)

[4.8 Lifestyle and Precautions 32](#_Toc197603967)

[5. Study treatment 33](#_Toc197603968)

[5.1 Study Medication and Administration 33](#_Toc197603969)

[5.2 Dose Adjustment of Study Medication 34](#_Toc197603970)

[5.3 Overdose of Study Medication 35](#_Toc197603971)

[5.4 Drug Interactions 36](#_Toc197603972)

[5.5 Concurrent medications and treatments 36](#_Toc197603973)

[5.6 Supply, Packaging, Labeling, and Storage of Study Medication 37](#_Toc197603974)

[5.7 Receipt, Counting, Distribution, and Storage of Study Medication 38](#_Toc197603975)

[6. Screening and Treatment Period Assessments 38](#_Toc197603976)

[6.1 Demographic Characteristics 38](#_Toc197603977)

[6.2 Weight and Height 38](#_Toc197603978)

[6.3 Vital Signs and Physical Examination 38](#_Toc197603979)

[6.4 Medical History and Other Past Histories 38](#_Toc197603980)

[6.5 History of Non-Muscle-Invasive Bladder Cancer (NMIBC) 38](#_Toc197603981)

[6.6 HER2 (IHC) Testing of Tumor Tissue 39](#_Toc197603982)

[6.7 Previous Medication/Concurrent Medication and Treatment 39](#_Toc197603983)

[6.8 Electrocardiogram (ECG) 40](#_Toc197603984)

[6.9 Echocardiogram 40](#_Toc197603985)

[6.10 Laboratory Tests 40](#_Toc197603986)

[6.11 Cytology Examination (Urine Cytology) 40](#_Toc197603987)

[6.12 Pregnancy Test 40](#_Toc197603988)

[7. Safety Assessments 40](#_Toc197603989)

[7.1 Definitions 40](#_Toc197603990)

[7.2 Collection and Recording of Safety Information 42](#_Toc197603991)

[7.3 Adverse Event Assessment 45](#_Toc197603992)

[7.4 Adverse Event Assessment and Follow-up Time Limits and Frequency 46](#_Toc197603993)

[7.5 Serious Adverse Event Reporting 46](#_Toc197603994)

[7.6 Pregnancy Events 47](#_Toc197603995)

[8. Study Schedule 47](#_Toc197603996)

[8.1 Study Visit Assessment Schedule 47](#_Toc197603997)

[9. Statistical Analysis 52](#_Toc197603998)

[9.1 Statistical Analysis Plan 52](#_Toc197603999)

[9.2 Sample Size Estimation 52](#_Toc197604000)

[9.3 Analysis Populations 52](#_Toc197604001)

[9.4 Statistical Analysis Methods 53](#_Toc197604002)

[10. Data Collection and Management Responsibilities 54](#_Toc197604003)

[10.1 Study Record Retention 56](#_Toc197604004)

[11. Supporting Documents and Operational Considerations 56](#_Toc197604005)

[11.1 Regulations, Ethics 56](#_Toc197604006)

[12. References 58](#_Toc197604007)

[13. Appendix 1 59](#_Toc197604008)

[13.1 1973 and 2004 WHO Bladder Cancer Grading Systems 59](#_Toc197604009)

[13.2 2017 UICC TNM Staging 60](#_Toc197604010)

[13.3 Non-muscle-invasive bladder cancer (NMIBC) risk classification criteria 61](#_Toc197604011)

[14. Appendix 2. New York Heart Association Functional Classification 61](#_Toc197604012)

[15. Appendix 3. ECOG Performance Status 62](#_Toc197604013)

[16. Appendix 4. Reference Formulas 62](#_Toc197604014)

[17. Appendix 5. Dose Escalation During Maintenance Infusion 62](#_Toc197604015)

# Results

## Supplementary Table S1. Representativeness of Study Participants.

| Cancer type(s) /subtype(s) /stage(s) /condition | Non-muscle invasive bladder cancer, cTa/T1±CIS |
| --- | --- |
| Sex | Men are more commonly affected by bladder cancer, with the male-to-female ratio remaining relatively steady at approximately 4:1. The ratio of male and female in this study is 2:1. |
| Age | Bladder cancer more commonly affects older individuals, with an average age at diagnosis of 73 years and >90% of cases occurring in persons >55 years of age. The median age of the participants was 69 years (range: 35 to 72 years). |
| Race/ethnicity | The study was performed in Han Chinese, the major ethnicity of China. |
| Geography | The trial enrolled patients from single sites in China. From 2008 to 2019, there were 143,675 records of patients diagnosed with bladder cancer, with an age-standardized 5-year relative survival rate at 71.5%. |
| Other considerations | Cigarette smoking is the most prominent contributor to bladder cancer development in most countries, with ~50% of all cases linked to this risk factor. In this trail, 22.2% patients had cigarette smoking history. |
| Overall representativeness of this study | The age distribution of our study is similar to the age distribution of bladder cancer globally, median age of 69. This study reflects a representative cohort of the Han Chinese population.  33.3% of enrolled patients were female, and this imbalance might be caused by small sample size in this phase I study. The higher number of female patients also led to a decrease in the proportion of smokers. |

# Protocol

**An Open-Label, Single-Arm, Single-Center Clinical Study to Evaluate the Safety and Tolerability of Intravesical RC48 for Patients with HER2-Expressing High-Risk Non-Muscle-Invasive Bladder Cancer (NMIBC) Who Have Not Previously Received Bacillus Calmette-Guérin (BCG) or Are Unresponsive to BCG**

**Protocol PI:** Tianxin Lin, M.D., Ph.D./

**Protocol ID:** RCVDUCIIR009

**Version Number:** Version 2.0

**Version Date:** September 5, 2023

**Sponsor:** Sun Yat-sen Memorial Hospital, Sun Yat-sen University

## Abstract

| Study Title | An Open-Label, Single-Arm, Single-Center Clinical Study to Evaluate the Safety and Tolerability of Intravesical RC48 for Patients with HER2-Expressing High-Risk Non-Muscle-Invasive Bladder Cancer (NMIBC) Who Have Not Previously Received Bacillus Calmette-Guérin (BCG) or Are Unresponsive to BCG |
| --- | --- |
| Study Medication | Disitamab vedotin (RC48) |
| Study Objective | Evaluate the safety and tolerability of intravesical RC48 in patients with HER2-expressing high-risk NMIBC who have not previously received BCG or are unresponsive to BCG. |
| Study Endpoints | 1. Primary Endpoints:  - Incidence and severity of dose-limiting toxicities (DLT) and adverse events (AE).  1. Secondary Endpoints:  - Vital signs, physical examinations, laboratory tests, and other examinations. - Recommended phase 2 dose (RP2D) / maximum tolerated dose (MTD). |
| Study design | This study is an open-label, single-arm, single-center investigator-initiated clinical trial aimed at evaluating the safety and tolerability of intravesical RC48 in patients with HER2-expressing high-risk NMIBC who have not previously received BCG or are unresponsive to BCG.  Eligible subjects must have completed a standard TURBT procedure within 3 weeks prior to enrollment, removing all visible lesions, with a postoperative pathological diagnosis of NMIBC, and are classified as high risk (including very high risk) according to the "Chinese Bladder Cancer Diagnosis and Treatment Guidelines (2022)". All subjects' surgical tumor specimens undergo HER2 testing, indicating HER2 expression, defined as immunohistochemistry (IHC) 1+, 2+, or 3+.  Included subjects are divided into two populations based on previous BCG treatment. One for high-risk NMIBC patients who have not previously received BCG, including the following situations: refusal of BCG treatment, contraindications for BCG use, or inaccessibility of BCG. The other for high-risk NMIBC patients who are unresponsive to BCG, meeting any of the following: persistent/recurrence of high-risk NMIBC within 12 months (±1 month) after completing adequate BCG treatment, or high-grade T1 disease at the first evaluation after induction BCG treatment.  Adequate BCG treatment is defined as: at least 5 BCG intravesical instillations completed within 2 months, followed by at least 2 BCG intravesical instillations within any continuous 6-week period within the next 10 months, i.e., at least "5+2" BCG intravesical instillations completed within approximately 12 months.  This study is in the dose-escalation phase, exploring the safety and tolerability of intravesical RC48, determining the maximum tolerated dose (MTD) of RC48 and the recommended Phase 2 dose (RP2D), thus providing a reference for a reasonable dosing regimen for subsequent studies.  A total of three dose groups are designed as 60 mg, 120 mg, and 180 mg, using the "3+3" dose-escalation method to include subjects. Eligible subjects receive intravesical RC48 once a week for 6 consecutive weeks. For patients receiving treatment, if there is no persistent/recurrence of NMIBC, disease progression, or intolerable toxicity, they can obtain RC48 for maintenance treatment free for up to one year. The medication plan for maintenance instillation treatment is once every 4 weeks, for a total of 11 times. Based on the individual response and tolerability of the subjects, open-dose-level maintenance treatment is adopted, and dose escalation can be considered (see Appendix 5 Dose Escalation During Maintenance Instillation).  At the same time, the safety and tolerability within the first 28 days after the first administration of the dose-limiting toxicity (DLT) assessment period are evaluated. According to the data of all subjects in the previous dose group who have completed the DLT assessment period, a comprehensive analysis is made to decide whether to start the enrollment of the next dose group.  The "3+3" principle adopted for the dose-escalation steps is as follows:   1. The first cohort of 3 eligible subjects starts with the 60 mg dose group. If no dose-limiting toxicity is observed in the 3 subjects during the DLT assessment period, the next dose group begins administration. If 1/3 of the subjects (1 case) exhibits DLT, an additional 3 subjects are added to this dose level for continued observation. If no DLT occurs in the additional 3 subjects, the dose can be escalated to the next group. If ≥1 case of DLT is observed among the additional 3 subjects, the dose escalation is halted, and the study retreats to the previous dose level (if the current group is 60 mg, it is reduced to 30 mg). 2. When escalating to 180mg, the investigators judge whether to escalate to 240mg or higher doses, as well as the most reasonable increment, based on the safety and tolerability information obtained during the study. The dose-escalation trial continues until the maximum tolerated dose (MTD) is reached, or the investigators terminate the escalation dose based on the known safety and efficacy characteristics from the escalation phase.   Safety during the study is assessed according to the NCI-CTCAE V5.0 standard, with observation indicators including vital signs, physical examination, laboratory tests, electrocardiogram, and echocardiogram, adverse events, and serious adverse events. |
| Study participants | Patients with HER2-expressing high-risk NMIBC who have not previously received BCG or are unresponsive to BCG. |
| Inclusion criteria | 1. Voluntarily agree to participate in the study and sign the informed consent form. 2. Male or female, aged 18-75 years (inclusive of both ages). 3. Disease criteria:  - High-risk NMIBC (including very high-risk group) referring to the Chinese Bladder Cancer Diagnosis and Treatment Guidelines (2022) (see Appendix 14.3 of the protocol for non-muscle-invasive bladder cancer risk classification standards), and also meet the condition of being primarily urothelial carcinoma. - Complete TURBT within 3 weeks before the treatment and all visible lesions have been removed; - Clinical staging with cTa/T1±CIS, N0, M0. No distant metastasis assessed by imaging within 3 months before enrollment.  1. Previous BCG treatment status meets one of the following two situations:  - High-risk NMIBC patients who have not previously received BCG treatment, including one of the following situations:  1. The patient refuses BCG treatment. 2. There are contraindications for BCG treatment. 3. BCG is not accessible.  - High-risk NMIBC patients who are unresponsive to BCG, meeting any of the following:  1. Persistent/recurrence of high-risk NMIBC within 12 months (±1 month) after completing adequate BCG treatment. 2. High-grade T1 at the first evaluation after induction treatment of BCG.   Note: Adequate BCG treatment is defined as at least 5 intravesical BCG treatments completed within 2 months, followed by at least 2 intravesical BCG treatments within any continuous 6-week period within the next 10 months.   1. Unsuitable for radical cystectomy assessed by urologist, or refuses radical cystectomy. 2. The patient's tumor tissue specimen obtained by TURBT has HER2 1+, 2+, or 3+ tested by immunohistochemistry (IHC). 3. ECOG performance status 0-2. 4. Adequate heart, bone marrow, liver, and kidney function, meeting the following criteria within 7 days before the treatment:  - Left ventricular ejection fraction ≥50%. - Hemoglobin ≥9g/dL. - Absolute neutrophil count (ANC) ≥1.5×10^9^/L. - Platelet count ≥100×10^9^/L. - Serum total bilirubin ≤1.5 times the upper limit of normal (ULN). - ALT and AST ≤2.5×ULN. - Blood creatinine ≤1.5×ULN or creatinine clearance (CrCl) ≥50mL/min calculated by the Cockcroft-Gault formula.  1. Female patients should be surgically sterilized, postmenopausal patients, or agree to use at least one medically recognized contraceptive method (such as intrauterine devices, oral contraceptives, or condoms) during the study treatment period and for 6 months after the end of the study treatment. 2. A blood pregnancy test negative within 7 days before enrollment, and false-positive results can be excluded by the investigator after ruling out pregnancy. Male patients should agree to use at least one medically recognized contraceptive method during the study treatment period and for 6 months after the end of the study treatment. 3. Willing and being able to comply with the trial and follow-up procedures. |
| Exclusion criteria | 1. MIBC (T2-4) and/or with regional lymph node or distant metastasis. 2. Combined with extravesical (i.e., urethra, ureter, or renal pelvis) urothelial carcinoma. 3. Received any other antitumor treatment within 4 weeks before study treatment, such as chemotherapy, radiotherapy, targeted therapy, immunotherapy, excluding immediate instillation chemotherapy completed after TURBT. 4. Had not recovered to grades 0-1 (CTCAE 5.0) from adverse events caused by previously used antitumor drugs within 2 weeks before starting the study treatment. 5. Scheduled for major surgery within 4 weeks before the start of the study treatment or during the trial period. 6. Serum virology tests (based on the reference values of the research center):  - Positive results for HBsAg or HBcAb, with concurrent detection of HBV DNA copies. - Positive result for HCVAb, with concurrent positive result for HCV RNA. - Positive result for HIVAb.  1. Received live vaccines within 4 weeks before the start of the study treatment or planned to receive any vaccines during the study period (except for inactivated novel coronavirus vaccines). 2. New York Heart Association (NYHA) class 3 or higher heart failure. 3. Serious arterial/venous thrombotic events or cardiovascular and cerebrovascular accidents within 6 months before the study treatment, such as deep vein thrombosis, pulmonary embolism, cerebral infarction, cerebral hemorrhage or myocardial infarction, excluding asymptomatic lacunar infarction that did not require clinical intervention. 4. Active or progressive infections requiring systemic treatment, such as active tuberculosis. 5. Active, uncontrolled systemic diseases or severe comorbidities judged by the investigator, including diabetes, hypertension, liver cirrhosis, interstitial pneumonia, obstructive pulmonary disease, etc. 6. Suffering from any other diseases, metabolic abnormalities, physical examination abnormalities, or laboratory test abnormalities, which led the investigator to suspect that the patient had a certain disease or condition that was not suitable for using the study treatment, or would affect the interpretation of the study results, or would put the patient at high risk. 7. Active autoimmune diseases requiring systemic treatment (such as the use of immunomodulatory drugs, corticosteroids, or immunosuppressants) within 6 months before the start of the study treatment, allowing for related replacement therapies (such as thyroid hormone replacement for adrenal or pituitary insufficiency, or physiological corticosteroid replacement therapy). 8. History of other malignancies within 5 years before the start of the study treatment, except for the following situations:  - Malignancies that could be expected to recover after treatment (including but not limited to thyroid cancer, cervical carcinoma in situ, basal or squamous cell skin cancer, or breast ductal carcinoma in situ treated with radical surgery). - Patients with prostate cancer who had undergone specific treatments (surgery or radiotherapy):  1. Stage T2N0M0 or earlier. 2. Gleason score ≤7 and prostate-specific antigen (PSA) undetectable for at least 1 year after anti-androgen therapy. 3. Patients who could receive specific treatment or were under active surveillance without specific treatment, with stable disease for 1 year before study enrollment. 4. History of allogeneic hematopoietic stem cell transplantation or organ transplantation. 5. Known allergy to RC48 or any of its components, or any excipients. 6. Pregnant or breastfeeding women. 7. Insufficient estimated patient compliance with participation in this clinical study. |
| Sample size | It is initially estimated that about 9 subjects are needed to determine the MTD, and subjects with unassessable DLT will be supplemented. The actual number of subjects receiving treatment may vary depending on the occurrence of adverse reactions during the study, which may result in more subjects being enrolled than initially anticipated. |
| Statistical analysis | All statistical analyses for this study will use SAS 9.4 or later versions. Descriptive statistics for continuous variables will include the number of cases, mean, standard deviation, median, minimum, and maximum values. Categorical variables will be described using frequency and percentage.  Summarize the occurrence of dose-limiting toxicities (DLT) in each dose group to evaluate the maximum tolerated dose (MTD). According to each dose group, statistics for TEAE, adverse reactions (AEs) that are definitely related, likely related, and possibly related to the test drug , serious adverse events, serious adverse reactions, adverse events or reactions with a severity of NCI-CTCAE (version 5.0) ≥3, etc. For vital signs, physical examinations, laboratory tests, and 12-lead electrocardiograms, provide a table of relative baseline changes or clinical significance relative to baseline changes. |

1. **Background**
   1. **Introduction**

Bladder cancer is the most common malignant tumor in the urinary system, with an incidence rate that ranks first among malignant tumors of the urinary system. Worldwide, the incidence rate of bladder cancer in males is 9.0 per 100,000, and in females, it is 2.2 per 100,000, respectively ranking 7th and 18th among malignant tumors in both sexes.^1^ In 2015, the incidence rate of bladder cancer in China was 5.8 per 100,000, with a male incidence rate of 8.8 per 100,000 and a female incidence rate of 2.6 per 100,000, ranking 7th and 16th among malignant tumors in males and females, respectively. Among patients with bladder cancer, smokers account for about 50% of the cases, with a risk increase of 2-3 times, and the risk is directly proportional to the intensity and duration of smoking. Approximately 20% of bladder cancer patients have an occupational exposure to carcinogens, and advanced age is an independent risk factor for bladder cancer. In addition, certain past treatment histories (including cyclophosphamide chemotherapy, pelvic radiotherapy, etc.), as well as genetic and gene abnormalities, are risk factors for bladder cancer, including oncogenes related to bladder cancer such as HER2, HRAS, BCL2, FR3, etc.

According to whether the tumor infiltrates the bladder muscle layer, using the 2017 TNM staging criteria of the International Union Against Cancer (UICC), bladder cancer is divided into non-muscle-invasive bladder cancer (NMIBC) and muscle-invasive bladder cancer (MIBC). NMIBC accounts for about 70%-80% of bladder cancer cases, including Ta stage (70%-75%), T1 stage (20%-25%), and Tis stage (carcinoma in situ [CIS], 5%-10%). The characteristics of NMIBC are high incidence and recurrence rates (20%-70%, depending on the risk level), requiring long-term cystoscopic monitoring, urine cytology examination, imaging examination, and bladder instillation chemotherapy or immunotherapy. MIBC accounts for about 25% of bladder cancer cases, with a 5-year mortality rate of about 40%-50% for diagnosed patients, significantly increasing patient treatment costs and mortality.^2^ According to the WHO 1973/2004 pathological grading combined with the 2017 TNM staging of UICC, NMIBC patients are divided into low-risk, intermediate-risk, and high-risk groups based on different recurrence and progression risks and prognoses. Forty percent of high-risk NMIBC will progress to muscle-invasive tumors, and high-grade NMIBC is more likely to progress, with nearly 75% of high-grade NMIBC recurring, progressing, or causing death within 10 years after diagnosis.

The two main principles of NMIBC treatment are: first, visible tumor lesions, especially T1 stage lesions, should be completely resected; second, bladder instillation treatment to eliminate minor residual lesions. The current main treatment for high-risk NMIBC is transurethral resection of bladder tumor (TURBT), but the tumor recurrence rate within 12 months after simple TURBT surgery is 40%-80%, and the disease progression rate within 24 months is as high as 20%-50%. The recurrence and progression of NMIBC may be related to tumor cell implantation, new tumors, or incomplete resection of the primary tumor. Repeat TURBT is recommended for some patients with indications. Bladder instillation is the main treatment method to prevent tumor recurrence and progression after TURBT surgery, and the drugs mainly include immunological agents (such as bacillus Calmette-Guérin [BCG] and interferons, etc.) and chemotherapy drugs (such as gemcitabine, mitomycin, and epirubicin, etc.). For high-risk NMIBC patients, it has been found that the 1-year recurrence-free survival rate or DFS rate of initial treatment of high-risk NMIBC patients with BCG instillation and chemotherapy instillation is 70%-90% and 50-70%, respectively. BCG instillation is more advantageous than single chemotherapy drugs in reducing disease recurrence and progression. Therefore, domestic and foreign guidelines recommend BCG bladder instillation as the standard treatment after TURBT for high-risk NMIBC patients. The BCG instillation scheme usually consists of 6 induction instillations, followed by maintenance treatment, and the duration of maintenance treatment has not yet reached a consensus, about 1-3 years. After BCG treatment, although the initial response rate is high, the response time often cannot be sustained, with 50%-77% of patients recurring within 5 years, and even reports of about 50% of patients recurring within 1 year after BCG treatment.^3^ Among them, about 20% of high-grade T1 stage patients can progress to muscle-invasive bladder cancer or metastatic disease within 5 years. In addition, 20% of patients are refractory to BCG, that is, they do not respond to initial BCG treatment. Compared with chemotherapy instillation, BCG instillation causes a significant increase in local and systemic adverse reactions, including local adverse reactions such as difficulty in urination (60%), bladder irritation (60%), chemical cystitis (30%), hematuria (20%), urinary tract infection (15%), and bladder contraction (3%), and systemic adverse reactions including fever, chills, and flu-like symptoms (30%), etc. In a randomized study of BCG maintenance treatment for 3 years, due to the high incidence of adverse reactions, a considerable proportion of patients discontinued BCG treatment due to intolerance, and only 16% of patients completed the planned medication plan.^4^ Based on the treatment effect and adverse reactions of BCG, BCG recurrence, BCG refractoriness, and BCG intolerance are considered as BCG treatment failure, among which BCG recurrence and BCG refractoriness belong to BCG non-response. The treatment of BCG non-responsive patients is recommended by the American Urological Association (AUA)^5^ and the European Urological Association (EUA) to undergo radical cystectomy or re-BCG treatment. However, the 1-year response rate of re-BCG treatment is only about 15%-20%, and these patients face a huge challenge in preventing recurrence and progression, especially in BCG refractory patients, where 50% of patients progress to MIBC within 5 years. Therefore, although BCG is recognized as the preferred drug for bladder instillation in high-risk NMIBC patients, there are clinical application deficiencies that limit its clinical use. Firstly, the incidence of adverse reactions to BCG treatment is high, and patients discontinue treatment due to intolerance, leading to poor efficacy. Meta-analysis found that patients with insufficient BCG instillation courses do not benefit more than chemotherapy instillation. Secondly, the drug is in short supply and expensive, and it has not been widely used in clinical practice in China at present. Thirdly, up to 40% of patients do not respond to BCG treatment, and the response rate to re-treatment is low, and the risk of recurrence and progression is significantly increased^6^.

At present, only two drugs have been approved by the FDA for high-risk NMIBC patients with CIS with or without papillary tumors who are unresponsive to BCG, unsuitable for or refuse cystectomy. One is valrubicin, approved in 1998, based on a multicenter phase II trial involving 90 patients who failed BCG treatment, with a study result of a 6-month complete response rate of 21% and a 2-year disease-free survival rate of 8%, and a median duration of response of 12 months.^7^ The other is the PD-1 inhibitor pembrolizumab, approved in 2020, based on the results of the phase II KEYNOTE-057 trial (NCT02625961), which is a single-arm, multicenter, open-label phase II study involving 148 high-risk NMIBC patients, of which 96 had CIS with or without papillary tumors. The results showed that 96 high-risk NMIBC patients with CIS at baseline had a 3-month complete remission rate of 41%, a median duration of response of 16.2 months, and 46% of patients with remission had a complete remission that lasted at least 12 months.^8^ N-803 (Anktiva), an IL-15 superagonist immune-stimulating complex that promotes the proliferation and activation of NK cells and CD8+ T cells, submitted a marketing application to the FDA in May 2022 for combined use with BCG, with the same indication for BCG-unresponsive CIS with or without papillary tumors. The application is based on data from cohort A (CIS with or without papillary tumors) of an open, single-arm, multi-cohort phase II/III clinical study (QUILT3.032 study, NCT03022825), which included 82 patients treated with N-803+BCG bladder instillation. The study results showed a complete remission rate of 71% (59/82), a median duration of response of 26.6 months, and 61.6% of patients with complete remission lasting at least 12 months. In addition, PK data showed that N-803 was only distributed in the bladder and no systemic absorption of N-803 was found. However, since this treatment method is combined with BCG, it is still not applicable to people who are unsuitable for, refuse BCG, or have no access to BCG.

Enfortumab vedotin, an ADC conjugated with MMAE targeting Nectin-4, like RC48, has been approved for the indication of advanced urothelial carcinoma (UC). Preclinical in vitro and in vivo studies have confirmed the antitumor activity of Enfortumab vedotin against human bladder cancer cell lines expressing Nectin-4. In cytotoxicity studies simulating bladder instillation drug exposure, it was found that compared with the small molecule MMAE, Enfortumab vedotin has stronger toxicity to tumor cells. In NMIBC orthotopic xenograft mouse model studies, the antitumor activity mediated by Enfortumab vedotin was confirmed by marked tumor bioluminescence imaging and IHC detection of Nectin-4-expressing tumor cells. In addition, the expression of Enfortumab vedotin in tumor cells further confirmed that Enfortumab vedotin can be absorbed by tumors. Repeated dose toxicity studies found that Enfortumab vedotin bladder instillation repeated dosing had good tolerability, with very small local toxicity and no systemic toxicity found at doses up to six times the maximum tolerated intravenous dose. The lack of systemic toxicity is related to very low systemic exposure (less than 1% of the equivalent intravenous dose C_max_) and MMAE not detected in the blood. In addition, the study results showed that the increase in MMAE levels in mouse bladder tissue is related to the increase in the total dose and concentration of Enfortumab vedotin, rather than changing the instillation volume or retention time. These study results provide a basis for further clinical studies of bladder instillation of Enfortumab vedotin in NMIBC patients.^9^ A phase I clinical study assessing the safety, tolerability, and dose expansion of Enfortumab vedotin bladder instillation in high-risk NMIBC patients unresponsive to BCG with CIS with or without papillary tumors (NCT05014139) is underway, and no relevant results have been released.

Current guidelines recommend radical cystectomy for high-risk NMIBC patients who are unsuitable for BCG, refuse BCG, or have no access to BCG, and who are unresponsive to BCG, with no standard treatment recommendations. However, surgery is highly traumatic for patients, and some elderly and physically unfit patients cannot tolerate it. Moreover, postoperative urinary diversion surgery is required, which severely affects the patient's quality of life. In addition, the incidence of severe perioperative complications of radical cystectomy is about 45%-70%,^10^ and the 90-day mortality rate after surgery is about 4%,^11^ and many patients refuse or are not suitable for cystectomy.

Therefore, for high-risk NMIBC patients who are unsuitable, refuse, or have no access to BCG, and who are unresponsive to BCG, and who are unsuitable or refuse radical cystectomy, the current available adjuvant treatments are very limited, and there is a huge and unmet clinical demand for safe and effective treatment methods. There is an urgent need for new safer and more effective alternative treatment plans, such as antibody-drug conjugates (ADCs) based on targeted specificity and high antitumor efficacy, to reduce the risk of patient recurrence and progression, preserve bladder function, extend the time of bladder removal or reduce the rate of bladder removal, and improve patient treatment tolerance.

- 1. **Bladder Cancer and HER2**

Human epidermal growth factor receptor 2 (HER2) is a member of the EGFR/ErbB family, encoded by the ERBB2 gene. Mutations in the ERBB2 gene leading to gene amplification and upregulation of HER2 protein have been found in various types of cancer, including bladder, breast, stomach, and lung. The abnormal increase in HER2 gene and protein expression leads to HER2-dependent increased cell proliferation, ultimately leading to the occurrence and development of cancer. Therefore, the persistent stimulation of cell proliferation by HER2 protein is considered a decisive factor in the carcinogenesis of HER2-positive tumor cells.

Numerous studies have shown that the expression of HER2 is closely related to the occurrence and progression of bladder cancer. HER2 plays a significant role in the diagnosis, prognosis, and targeted therapy of bladder cancer, especially the overexpression of HER2, which is closely related to the progression and poor prognosis of urothelial carcinoma of the bladder (UCB). A retrospective study found that in patients with muscle-invasive bladder cancer (MIBC), HER2 overexpression (IHC2+/3+) was associated with poor overall survival (OS) and disease-free survival (DFS).^12^ Additionally, HER2 expression has been proven to be associated with drug resistance in bladder cancer cells,^13^ enhanced metastasis and invasion,^14^ high tumor staging and grading,^15^ and tumor recurrence.^16^ HER2 is also an important target marker for various urothelial carcinoma targeted therapies, and the efficacy and safety of HER2 inhibitors in locally advanced and metastatic urothelial carcinoma have been confirmed in many preclinical and clinical studies.

As an early form of bladder cancer, non-muscle-invasive bladder cancer (NMIBC), research has found that 10%-40% of NMIBC patients have positive HER2 protein expression, and about 10% have HER2 gene alterations (including driver mutations and amplifications). A study analyzed the TURBT specimens from 83 patients with high-grade T1 bladder cancer and found that 21 (25%) were HER2 3+, 28 (34%) were HER2 2+, 26 (31%) were HER2 1+, and 8 were HER2 negative.^17^ A retrospective study found that HER2 gene alterations occurred more frequently in patients with high-grade tumors compared to those with low-grade tumors (13% vs. 3.8%), suggesting that HER2 gene alterations are associated with later stages of the disease and higher grades of histopathological types. Research has found that HER2 expression is an independent risk factor for recurrence-free survival (RFS) and progression-free survival (PFS) in NMIBC patients, indicating that HER2 expression is related to the recurrence and progression of NMIBC.^16,17^ Therefore, abnormal HER2 expression may be related to the risk stratification and poor prognosis of NMIBC.

- 1. **RC48 (Disitamab vedotin)**

Disitamab vedotin (RC48), independently developed by RemeGen, Ltd., is an antibody-drug conjugate (ADC) consisting of three parts: anti-human epidermal growth factor receptor 2 extracellular domain (HER2 ECD) antibody, linker (MC-Val-Cit-PAB), and the cytotoxic drug monomethyl auristatin E (Monomethyl Auristatin E, MMAE).

RC48 has multiple tumor cell-killing and anti-tumor effects. After the antibody part of RC48 binds to the extracellular domain of HER2 on the cell surface, the ADC complex is internalized by the cell and transported to the lysosome, where the linker is enzymatically cleaved to release the microtubule inhibitor MMAE, disrupting the intracellular microtubule network. This primarily manifests as microtubule disassembly, inducing cell cycle arrest at the G2/M phase, leading to mitotic cell cycle cessation and apoptosis. The released MMAE diffuses into the tumor microenvironment, causing bystander cytotoxicity in neighboring dividing cells. RC48 interferes with cellular transcription, growth, and proliferation by inhibiting downstream signaling pathways activated by HER2 (such as PI3K/AKT). In addition, anti-tumor activity in HER2-expressing human xenograft models, including breast, ovarian, gastric, and non-small cell lung models, even in models resistant to trastuzumab and lapatinib.

The efficacy and safety of RC48 in treating locally advanced or metastatic urothelial carcinoma with HER2 overexpression in phase II clinical studies (RC48-C005/RC48-C009, see section 1.3.2) have been analyzed, demonstrating the therapeutic benefits and safety in this patient population.

- - 1. **Preclinical Study Results of RC48**

Preclinical pharmacodynamic studies have shown that in vitro antitumor activity studies indicate that RC48 has dual effects of inhibiting the HER2 signaling pathway and inducing microtubule disassembly, exhibiting significant selective antitumor activity against HER2-positive tumor cells. RC48 can selectively inhibit the proliferation of HER2-positive tumor cells, induce cell cycle arrest, and apoptosis, and has an inhibitory effect on the proliferation of various HER2-positive bladder cancer cell lines, which is positively correlated with the expression level of HER2 and is concentration-dependent. In vivo antitumor studies have shown significant efficacy against HER2-positive human tumor xenografts in nude mice, especially for breast cancer resistant to trastuzumab and lapatinib, with a therapeutic effect significantly stronger than that of trastuzumab and lapatinib; moreover, the effects of RC48 are stronger than those of the reference drug T-DM1 at equivalent doses; in addition, RC48 administered via bladder instillation has a significant tumor-suppressing effect in a bladder orthotopic cancer nude mouse model, and compared with RC48 and buffer solution, a high dose of RC48 (12.5mg/kg) can effectively extend the survival period of tumor-bearing nude mice. The in vivo anti-HER2-positive tumor effect of RC48 is significantly stronger than that of an equivalent amount of naked antibody and conjugated small molecule MMAE used in combination.

Long-term toxicity studies have shown that after seven consecutive administrations of RC48 Q2W, the main toxic reactions in SD rats and crab-eating macaques were immunosuppression and hematopoietic suppression, with rats also showing gastrointestinal damage and atrophy of the testes and epididymis, with maximum tolerated doses (MTD) of 12mg/kg and 10mg/kg, respectively; the toxic reactions of RC48 are essentially consistent with those of MMAE. Experiments on bladder instillation for bladder orthotopic cancer showed no significant abnormalities in routine blood tests and blood biochemistry in tumor-bearing nude mice after RC48 bladder instillation, and there was no significant weight loss at high doses (12.5mg/kg) compared to before administration, with less cachexia.

Immunotoxicity and/or immunogenicity tests have shown that RC48 has an immunosuppressive effect; after repeated intravenous administration, the incidence of immunogenic anti-drug antibodies (ADA) positive in SD rats increases with the increase of the dose, while in crab-eating macaques, it decreases with the increase of the dose, which may be related to its immunosuppressive effect in crab-eating macaques.

For specific preclinical study results and data, please refer to the investigator's manual and related experimental materials.

- - 1. **Clinical Study of RC48 In Treatment of Advanced Bladder Cancer**

In 2015, the National Medical Products Administration approved the clinical trial of Renviciclib (clinical trial permit number: 2015L02291) conducted by RemeGen, Ltd.

The safety and efficacy of RC48 in treating locally advanced or metastatic urothelial carcinoma with HER2 overexpression have been confirmed in clinical studies. RC48-C005 is a single-arm, open-label, multicenter Phase II clinical study evaluating the efficacy of RC48 in patients with locally advanced or metastatic HER2-overexpressing urothelial carcinoma of the bladder. A total of 43 patients with HER2-overexpressing (IHC2+ or 3+) locally advanced or metastatic urothelial carcinoma who had previously received ≥1 line of systemic chemotherapy were enrolled in this study. According to the IRC assessment, the objective response rate (ORR) was 51.2% (95% CI: 35.5, 66.7), the disease control rate (DCR) was 90.7% (95% CI: 77.9, 97.4), the median duration of response (DOR) was 7.0 months (95% CI: 4.7, 12.4), the median progression-free survival (PFS) was 6.9 months (95% CI: 5.4, 9.0), the median overall survival (OS) was 13.9 months (95% CI: 9.1, NE), the 1-year survival rate was 55.8%, and the 2-year survival rate was 38.6%. RC48 demonstrated a significant improvement in objective response rate and showed good survival benefits in patients with locally advanced or metastatic urothelial carcinoma who had previously received ≥1 line of systemic chemotherapy, compared to historical data.

RC48-C009 is an open-label, single-arm, multicenter Phase II clinical study evaluating the efficacy and safety of RC48 in patients with locally advanced or metastatic HER2-overexpressing urothelial carcinoma who had failed treatment with gemcitabine, platinum, and taxane. A total of 64 patients with HER2-overexpressing (IHC2+ or 3+) locally advanced or metastatic urothelial carcinoma were enrolled in this study. Eligible subjects received RC48-ADC at a dose of 2.0mg/kg Q2W until disease progression, intolerable toxicity, or withdrawal. The primary endpoint of the study was ORR, and secondary endpoints included PFS, DOR, DCR, OS, and safety. In the full analysis set (FAS) population of 64 patients, the ORR assessed by IRC was 50.0% (95% CI: 37.2, 62.8), the median DOR was 8.3 months (95% CI: 4.3, 12.0), the median PFS was 5.1 months (95% CI: 4.0, 6.9), and the median OS was 14.2 months (95% CI: 8.7, 19.2).

Based on the above RC48-C005 and C009 clinical studies, RC48 was conditionally approved by the National Medical Products Administration in December 2021 for the treatment of patients with locally advanced or metastatic urothelial carcinoma who had previously received platinum-containing chemotherapy and had HER2 overexpression.

RC48-C014 is a single-arm, open-label, single-center Ib/II phase clinical study initiated by the investigator, aimed at exploring the efficacy and safety of RC48 combined with toripalimab injection for the treatment of locally advanced or metastatic urothelial carcinoma. Safety results indicate that the combination of RC48 and toripalimab has good safety and tolerability. The safety profile is similar to that of the single-agent clinical studies of RC48 and toripalimab, with no new safety risks observed.

- - 1. **Development Rationale for Intravesical RC48**

The structure of the bladder wall includes the urothelium, basement membrane, lamina propria (which contains microvessels and lymphatics), muscular layer, and serosal layer. These structures possess intercellular ion channels and tight junctions that prevent the absorption of water by the bladder wall and form a barrier against hematuria. This barrier prevents the absorption of harmful toxins in the urine, such as urea and creatinine, and also prevents large molecules administered through bladder instillation from penetrating the bladder wall. Drugs that diffuse to the deep layers of the bladder wall are quickly cleared by capillaries, leading to a gradient decrease in drug concentration. Therefore, it is possible to control and limit the transfer of drugs administered through bladder instillation to the deep layers of the bladder wall. Additionally, the bladder wall tissue has strong compliance, allowing it to store urine. In this state, drugs administered within the bladder can maintain full contact with the bladder wall.

Due to the unique anatomical characteristics and physiological functions of the bladder, the pharmacokinetics of intravesical therapy are different and superior to oral and intravenous administration routes. In most cases, only a small portion of the drug reaches the bladder lesion through oral and intravenous administration routes due to poor oral absorption and systemic metabolism loss. In contrast, intravesical administration is a non-invasive route of drug delivery through a catheter, which can locally deliver high concentrations of anticancer drugs. The direct contact of the drug with the tumor aims to kill or induce apoptosis in tumor cells, potentially maximizing therapeutic efficacy by increasing local drug exposure and significantly reducing systemic exposure, thereby decreasing systemic adverse reactions. This has been confirmed with chemotherapy drug instillation, but conventional chemotherapeutic drugs have low tissue selectivity and require high doses to achieve anticancer effects, which can lead to extensive mucosal damage.

Therefore, there is a higher demand for drugs that can be administered locally, have high tumor tissue targeting, and cause minimal damage to normal tissues in the development of NMIBC treatments.

Multiple large molecule drugs, including pembrolizumab (a PD-1 inhibitor) and durvalumab (a PD-L1 inhibitor), have undergone clinical studies in high-risk NMIBC patients through both intravenous and bladder instillation administration routes. Currently, several ADC drugs, such as Vicinium and Enfortumab vedotin, have completed or are conducting multiple clinical studies treating high-risk NMIBC through bladder instillation.

RC48 enters the bladder through bladder instillation, and the antibody part can specifically bind to HER2-expressing tumor cells, entering the cells to release cytotoxic small molecule drugs. This targeted approach can specifically eliminate residual minimal lesions or implanted tumor cells without affecting the normal urothelial cells of the bladder wall. Preclinical studies have shown that intravesical instillation of RC48 in orthotopic bladder cancer nude mice can observe significant tumor suppression effects. Compared with epirubicin and buffer solution, a high dose of RC48 (12.5mg/kg) can effectively extend the survival period of tumor-bearing nude mice. Therefore, it is anticipated that RC48 bladder instillation treatment for high-risk NMBC can better prevent tumor recurrence and progression, reduce and avoid bladder resection, improve prognosis and survival quality, and cause less damage to the normal bladder mucosa. This has also been confirmed in the preclinical results of other ADC drugs, such as Enfortumab vedotin, which has demonstrated significant antitumor activity and good safety and tolerability of bladder instillation in mice with orthotopic bladder cancer.

As for the risk of systemic exposure, it has been confirmed that various drugs, including ADCs, various antibody large molecules, and small molecule drugs administered through bladder instillation have low levels of exposure in the blood, minimal systemic absorption, and blood drug concentrations far below the systemic exposure levels observed with equivalent doses of intravenous administration. Preclinical results of the ADC drug Enfortumab vedotin suggest that bladder instillation at doses up to six times the intravenous dose has minimal local adverse reactions and no observed systemic adverse reactions. The systemic exposure of Enfortumab vedotin at this dose is much lower than intravenous administration (bladder instillation <1% of the equivalent intravenous dose Cmax), and MMAE was not detected in the blood. The safety and tolerability of the intravenous dose of Enfortumab vedotin, which has been approved for the treatment of urothelial carcinoma, have been confirmed. Large molecule drugs such as pembrolizumab and N-803, when administered through the bladder, have not detected drug concentrations in the patient's blood (below the lower limit of detection), and the drug distribution is limited to the bladder.^18^ Small molecule chemotherapeutic drugs commonly used in clinical bladder instillation, including mitomycin, gemcitabine, and epirubicin,^19,20^ have blood drug concentrations far lower than those observed with similar systemic doses, and the drugs are quickly cleared. Several hours after instillation, the drug concentration in the plasma is undetectable, and even during the bladder retention period of the drug, the plasma drug concentration has decreased, and most of the drug can be recovered in the bladder. In terms of safety, animal experiments of RC48 bladder instillation for orthotopic bladder cancer have shown no significant abnormalities in routine blood tests and blood biochemistry in tumor-bearing nude mice after RC48 bladder instillation, and there was no significant weight loss at high doses (12.5mg/kg) compared to before administration, with less cachexia. The incidence of adverse reactions to commonly used chemotherapeutic drugs in clinical bladder instillation is significantly lower than that of intravenous administration, with lower severity and mainly local symptoms, most of which are reversible. Therefore, it is speculated that the intravesical administration of RC48 has low local toxicity, minimal systemic absorption, reduced systemic safety-related risks compared to intravenous administration, and increased patient tolerability.

Based on the effective tumor suppression and good safety of RC48 bladder instillation in preclinical models of HER2-expressing orthotopic bladder cancer, as well as the efficacy and safety data of intravenous RC48 in clinical studies of locally advanced or metastatic urothelial carcinoma with HER2 expression, and the preclinical studies of ADC drug Enfortumab vedotin showing significant antitumor activity and good safety and tolerability of bladder instillation for bladder orthotopic cancer, as well as the extremely low systemic exposure of ADC and small molecule toxin MMAE, it is anticipated that RC48 bladder instillation may be a more promising treatment method for high-risk NMBC at an early stage.

Therefore, this study plans to explore the efficacy and safety of intravesical RC48 in patients with high-risk NMIBC who have not previously received BCG or are unresponsive to BCG. Since there is currently no safety data on intravesical RC48 in humans, this study will use a dose-escalation approach to explore the safety and tolerability (MTD) of intravesical RC48 in patients with HER2-expressing high-risk NMIBC who have not previously received BCG or are unresponsive to BCG.

1. **Study Purpose and Endpoints**
   1. **Study Purpose**

To evaluate the safety and tolerability (MTD) of intravesical RC48 in patients with HER2-expressing, high-risk non-muscle-invasive bladder cancer (NMIBC) who have not previously received bacillus Calmette-Guérin (BCG) or are unresponsive to BCG.

- 1. **Study Endpoints**
     1. **Primary Endpoints**

Incidence and severity of dose-limiting toxicities (DLT) and adverse events (AE).

- - 1. **Secondary Endpoints**
- Vital signs, physical examinations, laboratory tests, and other examinations.
- Recommended Phase 2 Dose (RP2D) / Maximum Tolerated Dose (MTD).

1. **Study Design**
   1. **Overall Summary**

This study is an open-label, single-arm, single-center investigator-initiated clinical trial aimed at evaluating the safety and tolerability of intravesical RC48 for patients with HER2-expressing, high-risk NMIBC who have not previously received BCG or are unresponsive to BCG.

Eligible subjects must have completed a standard transurethral resection of bladder tumor (TURBT) procedure within 3 weeks prior to enrollment, removing all visible lesions, with a postoperative pathological diagnosis of NMIBC, and are classified as high risk (including very high risk) according to the "Chinese Bladder Cancer Diagnosis and Treatment Guidelines (2022)". All subjects' surgical tumor specimens undergo HER2 testing, indicating HER2 expression, defined as immunohistochemistry (IHC) 1+, 2+, or 3+.

Included subjects are divided into two populations based on previous BCG treatment. One for high-risk NMIBC patients who have not previously received BCG, including the following situations: refusal of BCG treatment, contraindications for BCG use, and inaccessibility of BCG. The other for high-risk NMIBC patients who are unresponsive to BCG, meeting any of the following: persistent/recurrence of high-risk NMIBC within 12 months (±1 month) after completing adequate BCG treatment, high-grade T1 disease at the first evaluation after induction BCG treatment.

Adequate BCG treatment is defined as: at least five BCG intravesical instillations completed within 2 months, followed by at least 2 BCG intravesical instillations within any continuous 6-week period within the next 10 months, i.e., at least "5+2" BCG intravesical instillations completed within approximately 12 months.

Patients entering the study will proceed to the dose-escalation phase to explore the safety and tolerability of intravesical RC48, determining the maximum tolerated dose (MTD) of RC48 and the recommended Phase 2 dose (RP2D), thus providing a reference for a reasonable dosing regimen for subsequent studies.

A total of three dose groups are designed as 60 mg, 120 mg, and 180 mg, using the "3+3" dose-escalation method to include subjects. Eligible subjects receive intravesical RC48 once a week for 6 consecutive weeks. For patients receiving treatment, if there is no persistent/recurrence of NMIBC, disease progression, or intolerable toxicity, they can obtain RC48 for maintenance instillation treatment free of charge for up to one year. The medication plan for maintenance instillation treatment is once every 4 weeks, for a total of 11 times. Based on the individual response and tolerability of the subjects, open-dose-level maintenance instillation treatment is adopted, and dose escalation can be considered (see Appendix 5 Dose Escalation During Maintenance Instillation).

Simultaneously, the safety and tolerability within the first 28 days after the first administration of the dose-limiting toxicity (DLT) assessment period are evaluated.

Investigators will make a comprehensive analysis based on the data from all subjects in the previous dose group who have completed the DLT assessment period to decide whether to proceed with the enrollment of the next dose group.

The "3+3" principle adopted for the dose-escalation steps is as follows:

1. The first cohort of 3 eligible subjects starts with the 60 mg dose group. If no dose-limiting toxicity is observed in the 3 subjects during the DLT assessment period, the next dose group begins administration. If 1/3 of the subjects (1 case) exhibits DLT, an additional 3 subjects are added to this dose level for continued observation. If no DLT occurs in the additional 3 subjects, the dose can be escalated to the next group. If ≥1 case of DLT is observed among the additional 3 subjects, the dose escalation is halted, and the study retreats to the previous dose level (if the current group is 60 mg, it is reduced to 30 mg).
2. When escalating to 180mg, investigators judge whether to escalate to 240mg or higher doses, as well as the most reasonable increment, based on the safety and tolerability information obtained during the study. The dose-escalation trial continues until the maximum tolerated dose (MTD) is reached, or the investigators terminate the escalation dose based on the known safety and efficacy characteristics from the escalation phase.

Safety during the study is assessed according to the NCI-CTCAE V5.0 standard, with observation indicators including vital signs, physical examination, laboratory tests, electrocardiogram, and echocardiogram, adverse events, and serious adverse events.

- 1. **Dose-Limiting Toxicity (DLT)**

Dose-limiting toxicity (DLT) is defined as any of the following toxicity reactions that occur during the DLT evaluation period (from the first administration to 28 days after administration) and are considered by the investigator to have a reasonable association with RC48-ADC administration, according to the 5-level grading standard of NCI-CTCAE V5.0:

1. Grade 4 neutropenia lasting more than 3 days after treatment with granulocyte colony-stimulating factor (G-CSF); or grade 4 neutropenia re-emerging after recovery to normal levels with G-CSF treatment.
2. Grade 3 neutropenic fever (defined as an absolute neutrophil count [ANC] <1000/mm3 with fever, body temperature greater than 38.3℃ or persistent fever, body temperature sustained higher than 38°C for more than 1 hour).
3. Grade 3 neutropenia with evidence of infection.
4. Grade 3 or higher thrombocytopenia.
5. Grade 3 or higher non-hematologic toxicity, except for the following situations:

- Grade 3 nausea, vomiting, or diarrhea controlled with appropriate treatment and returned to grade 1 within 7 days.
- Grade 3 rash controlled with appropriate treatment and returned to grade 1 within 7 days.
- Grade 3 fatigue controlled with appropriate treatment and returned to grade 1 within 7 days.
- Grade 3 fever (without neutropenia) controlled with appropriate treatment and returned to ≤ grade 2 within 7 days.
- Grade 3 or higher asymptomatic serum creatine kinase elevation (i.e., not accompanied by signs, symptoms, or other laboratory abnormalities associated with rhabdomyolysis or myocardial injury), considered by the investigator to be clinically insignificant and returned to ≤ grade 2 within 14 days after treatment discontinuation;
- Grade 3 laboratory test abnormalities, but without clinical symptoms and considered by the investigator to have no clinical significance.

1. Grade 3 or higher urinary retention lasting 72 hours or more.
2. Grade 3 or higher frequency of urination lasting 72 hours or more.
3. Grade 3 or higher hematuria lasting 72 hours or more.
4. Grade 3 or higher cystitis lasting 72 hours or more.
5. Grade 3 or higher urinary urgency or increased nocturia lasting 72 hours or more.

DLT Population Definition: Subjects who have received at least one intravesical instillation of RC48-ADC during the DLT evaluation period and have undergone adequate safety assessment, and have been observed for at least 28 days after the first administration, unless the subject discontinues study treatment due to DLT.

- 1. **Maximum Tolerated Dose (MTD)**

The maximum tolerated dose (MTD) is defined as the highest dose level at which the incidence of DLT is less than 1/3 within the first 28 days after the first administration.

- 1. **Dose Escalation Starting Dose and Expected Maximum Dose Determination Basis**

A non-clinical pharmacodynamic study conducted by the investigator showed that the inhibition of bladder cancer cells by RC48 is dose-dependent. The efficacy and safety of intravesical RC48 in a bladder cancer orthotopic model showed that RC48 can effectively inhibit the growth of bladder cancer, with an effective dose of 2.5mg/kg (approximately 1mg/mL) to 12.5mg/kg (approximately 5mg/mL), and all nude mice in the high-dose group survived until the end of the study. Intravesical RC48 did not cause toxic side effects such as myelosuppression or liver and kidney function damage, and the safety was good.

Referencing the in vivo efficacy results, the recommended starting dose for human bladder instillation testing is 60mg (approximately 1mg/mL), with the dosing concentration being essentially equivalent to the in vivo effective concentration.

The in vivo efficacy results suggest that at high concentrations of 12.5 mg/kg (approximately 5 mg/mL), no toxicity related to systemic exposure was found. Repeated dose toxicity study results in nude mice showed that after seven administrations of 12.5 mg/kg (approximately 5 mg/mL), 25 mg/kg (approximately 10 mg/mL), and 50 mg/kg (approximately 20 mg/mL), no safety risks were observed in the low and medium dose groups, and no obvious organic changes were observed in various organs. In the high-dose group, 2 out of 6 mice died, and inflammation was observed in the bladder upon dissection. Based on the body surface area method, the equivalent human equivalent dose of the high-dose group is 240 mg. However, based on the concentration results, the safe concentration range is 1 mg/mL - 10 mg/mL. The effect of bladder instillation is related to both the concentration and dose of the drug. Among them, the concentration of the drug is more important than the drug dose. Considering the dose and concentration results of the preclinical efficacy and toxicity studies, the maximum recommended human dose is 180 mg (3.6 mg/mL) or 240 mg (4.8 mg/mL).

Since there is no more non-clinical data to support the maximum tolerated dose (MTD) of bladder instillation and the irritation to the bladder mucosa, considering the safety of the subjects, three dose groups are temporarily set with a maximum dose of 180mg, namely 60, 120, and 180mg. When escalating to 180 mg, the investigators judge whether to escalate to 240 mg or higher doses, as well as the most reasonable increment, based on the safety and tolerability information obtained during the study. The dose-escalation trial continues until the maximum tolerated dose (MTD) is reached, or the investigators terminate the escalation dose based on the known safety and efficacy characteristics from the escalation phase.

- 1. **Dosing Scheme Design Basis**

Referencing the "Urothelial Carcinoma Diagnosis and Treatment Guidelines (2022 CSCO)" for the recommended scheme of chemotherapy drug bladder instillation for intermediate and high-risk NMIBC, the intravesical RC48 scheme is once a week for 6 consecutive weeks.

For patients receiving treatment, if there is no persistent/recurrence of NMIBC, disease progression, or intolerable toxicity, they can obtain RC48 for maintenance treatment free of charge for up to one year. The medication plan for maintenance instillation treatment is once every 4 weeks, for a total of 11 times. Based on the individual response and tolerability of the subjects, open-dose-level maintenance instillation treatment is adopted, and dose escalation can be considered (see Appendix 5 Dose Escalation During Maintenance Instillation).

1. **Study participants**
   1. **Inclusion criteria**

Subjects must meet all of the following criteria for enrollment:

1. Voluntarily agree to participate in the study and sign the informed consent form.
2. Male or female, aged 18-75 years (inclusive of both).
3. Disease criteria:

- Subjects' NMIBC risk group classification meets the high-risk group (including very high-risk group), referring to the "Chinese Bladder Cancer Diagnosis and Treatment Guidelines (2022)" (see Appendix 14.3 for non-muscle-invasive bladder cancer risk classification standards), and also meets the condition of being primarily urothelial carcinoma.
- Completed TURBT within 3 weeks before study medication and all visible lesions have been removed.
- Clinical staging (cTa/T1±CIS, N0, M0) and no distant metastasis assessed by imaging within 3 months before enrollment;

1. Previous BCG treatment status meets one of the following two situations:

- High-risk NMIBC patients who have not previously received BCG treatment due to subjective or objective conditions, including refusing BCG treatment, contraindications for BCG treatment, and BCG is not accessible.
- High-risk NMIBC patients who are unresponsive to BCG, meeting any of the following: 1. Persistent/recurrence of high-risk NMIBC within 12 months (±1 month) after completing adequate BCG treatment; 2. High-grade T1 disease at the first evaluation after induction BCG treatment. Note: Adequate BCG treatment is defined as at least 5 BCG intravesical instillations completed within 2 months, followed by at least 2 BCG intravesical instillations within any continuous 6-week period within the next 10 months, i.e., at least "5+2" BCG intravesical instillations completed within approximately 12 months.

1. Assessed by a urologist as unsuitable for radical cystectomy for bladder cancer or refusing radical cystectomy.
2. Subjects' tumor tissue specimens obtained within 3 weeks after TURBT surgery have HER2 expression tested by immunohistochemistry (IHC) and meet 1+, 2+, or 3+.
3. ECOG performance status 0-2.
4. Adequate heart, bone marrow, liver, and kidney function, within 7 days before study medication should meet the following criteria (normal values based on the clinical trial center):

- Left ventricular ejection fraction ≥50%.
- Hemoglobin ≥9g/dL.
- Absolute neutrophil count (ANC) ≥1.5×109/L.
- Platelet count ≥100×109/L.
- Serum total bilirubin ≤1.5 times the upper limit of normal (ULN).
- ALT and AST ≤2.5×ULN.
- Blood creatinine ≤1.5×ULN or creatinine clearance (CrCl) ≥50mL/min calculated by the Cockcroft-Gault formula;

1. Female subjects should be surgically sterilized, postmenopausal patients, or agree to use at least one medically recognized contraceptive method (such as intrauterine devices, oral contraceptives, or condoms) during the study treatment period and for 6 months after the end of the study treatment. A blood pregnancy test within 7 days before enrollment must be negative, and false-positive results can be excluded by the investigator after ruling out pregnancy. Male subjects should agree to use at least one medically recognized contraceptive method during the study treatment period and for 6 months after the end of the study treatment.
2. Willing and able to comply with the trial and follow-up procedures.
   1. **Exclusion criteria**

Subjects meeting the following criteria cannot be included in this study:

1. Muscle-invasive bladder cancer (T2 and above) and/or with regional lymph node and distant metastasis.
2. Combined with extravesical (i.e., urethra, ureter, or renal pelvis) urothelial carcinoma.
3. Received any other antitumor treatment within 4 weeks before study medication, such as chemotherapy, radiotherapy, targeted therapy, immunotherapy, etc., excluding immediate instillation chemotherapy completed after TURBT.
4. Within 2 weeks before starting the study medication, has not recovered to CTCAE 5.0 grades 0-1 from adverse events caused by previously used antineoplastic medications.
5. Scheduled for major surgery within 8 weeks before the start of the study medication or during the trial period.
6. Serum virology tests (based on the reference values of the research center):

- Positive results for HBsAg or HBcAb, with concurrent detection of HBV DNA copies.
- Positive result for HCVAb, with concurrent positive result for HCV RNA.
- Positive result for HIVAb.

1. Received live vaccines within 4 weeks before the start of the study medication or plans to receive any vaccines during the study period (except for inactivated novel coronavirus vaccines).
2. New York Heart Association (NYHA) class 3 or higher heart failure.
3. Serious arterial/venous thrombotic events or cardiovascular and cerebrovascular accidents within 6 months before the study medication, such as deep vein thrombosis, pulmonary embolism, cerebral infarction, cerebral hemorrhage, myocardial infarction, etc., excluding asymptomatic lacunar infarction that does not require clinical intervention.
4. Active or progressive infections requiring systemic treatment, such as active tuberculosis.
5. Active, uncontrolled systemic diseases or severe comorbidities judged by the investigator, including diabetes, hypertension, liver cirrhosis, interstitial pneumonia, obstructive pulmonary disease, etc.
6. Suffering from any other diseases, metabolic abnormalities, physical examination abnormalities, or laboratory test abnormalities, which lead the investigator to suspect that the patient has a certain disease or condition that is not suitable for using the study medication, or will affect the interpretation of the study results, or will put the patient at high risk.
7. Active autoimmune diseases requiring systemic treatment (such as the use of immunomodulatory drugs, corticosteroids, or immunosuppressants) within 6 months before the start of the study medication, allowing for related replacement therapies (such as thyroid hormone replacement for adrenal or pituitary insufficiency, or physiological corticosteroid replacement therapy).
8. History of other malignant tumors within 5 years before the start of the study medication, except for the following situations:

- Malignant tumors that can be expected to recover after treatment (including but not limited to thyroid cancer, cervical carcinoma in situ, basal or squamous cell skin cancer, or breast ductal carcinoma in situ treated with radical surgery).
- Patients with prostate cancer who have undergone specific treatments (surgery or radiotherapy): a) Stage T2N0M0 or earlier; b) Gleason score ≤7 and prostate-specific antigen (PSA) undetectable for at least 1 year after anti-androgen therapy; c) Patients who can receive specific treatment or are under active surveillance without specific treatment, with stable disease for 1 year before study enrollment;

1. History of allogeneic hematopoietic stem cell transplantation or organ transplantation.
2. Known allergy to RC48-ADC or any of its components, or any excipients.
3. Pregnant or breastfeeding women.
4. Insufficient estimated patient compliance with participation in this clinical study.
   1. **Treatment Termination/Early Termination**

If a subject experiences any medical condition that the investigator believes may jeopardize the subject's safety if they continue to participate in the study, the investigator has the right to decide to terminate the subject's study treatment. All subjects who have signed the informed consent form and have been screened and qualified to enter the trial have the right to terminate study treatment at any time. For all subjects who discontinue study treatment, the reasons for discontinuation should be recorded in the electronic case report form (eCRF) and the original medical records, and unused study medication must be inventoried and returned. If a subject discontinues study treatment due to adverse events or abnormal clinical laboratory test results, they should continue to be followed up until the subject recovers or the disease stabilizes or can be explained.

During the study period, if a subject withdraws from medication treatment without withdrawing informed consent, they continue in the study (tumor assessment follow-up), completing the required visit procedures outlined in the protocol. If a subject discontinues study treatment and refuses to return to the research center for visits and/or tumor assessments, they are withdrawn from the study.

Subject withdrawal from treatment may include but is not limited to the following reasons. Once a subject withdraws from treatment, they are not allowed to continue treatment.

Subjects must terminate study treatment when the following conditions occur (whichever occurs first):

1. Completion of treatment as specified in the protocol.
2. Persistent/recurrent NMIBC, disease progression, initiation of new antitumor treatment, etc., as determined by cystoscopy, pathology, and/or imaging.
3. Death.
4. Inability to tolerate toxicity despite dose adjustments according to the protocol.
5. Request by the subject or their legal representative to terminate treatment.
6. The investigator considers it in the best interest of the subject to terminate study treatment.
7. Pregnancy during the study.
8. Subject is lost to follow-up.
9. Study is terminated prematurely.

Withdrawal from the Study

If a subject experiences any medical condition that the investigator believes may jeopardize the subject's safety if they continue to participate in the study, the investigator has the right to decide to withdraw the subject from the study. All subjects who have signed the informed consent form and have been screened and qualified to enter the trial have the right to withdraw from the study at any time. If a subject withdraws from the study, they will no longer receive treatment or undergo visits within the study.

Subjects must withdraw from the study under the following circumstances:

1. The subject or their legal representative requests to withdraw informed consent.
2. The subject is lost to follow-up.
3. The investigator believes the subject is no longer suitable for participation in the study.
   1. **Study Termination/Cessation**

The study is considered complete two years after the first treatment of the last subject. The investigator has the right to terminate the study at any time. Reasons for terminating the study include but are not limited to the following:

1. The number and severity of adverse events in this or other studies suggest that continuing the study poses a significant risk to subjects, outweighing the benefits.
2. The slow enrollment rate at the research center prevents the completion of the enrollment plan within the specified time.
3. The quality of existing clinical data is poor, which is not conducive to continuing subsequent research.
4. The study is terminated due to changes in regulations and policies of the national drug administration department.
5. Other reasons.
   1. **Subject Loss to Follow-up**

If a subject does not return to the research center for scheduled visits and the research center staff cannot contact them, the subject will be considered lost to follow-up.

If a subject does not return to the research center for the required study visits, the following actions must be taken:

1. The research center attempts to contact the subject, reschedule missed visits, explain the importance of adhering to the visit schedule, and confirm whether the subject is willing and/or should continue to participate in the study.
2. Before a subject is considered lost to follow-up, the investigator or designated personnel will make every effort to re-establish contact with the subject. These attempts to contact the subject should be recorded in the subject's medical records or study documents.
3. If the subject still cannot be contacted, he/she will be considered lost to follow-up and will be withdrawn from the study.
   1. **Subject Numbering and Name Abbreviation**

Subject numbers are five-digit numbers. The first 1-2 digits represent the research center number, and the 3-5 digits represent the sequence number of the subjects screened within the research center. For example, the fifth subject screened at the 01 center is assigned the subject number "01005".

Subject name pinyin abbreviations consist of four letters. For two-character names, the first two letters of the pinyin are filled in, and if the second character's pinyin has only one letter, the first character's first letter is filled in again; for three-character names, the initials of the first three characters and the second letter of the third character are filled in. If the third character's pinyin has only one letter, the initials of the first two characters and the initials of the third character are filled in; for four-character names, the initials of each character are filled in.

- 1. **Screening Failure**

Screening failure is defined as subjects who agree to participate in the clinical trial but are not included in the study for any reason (e.g., failure to meet the study population selection criteria). Information required to be recorded for subjects who fail screening includes demographic information, reasons for screening failure, and safety information after signing the informed consent form.

Based on the investigator's judgment, subjects who do not meet the study participation criteria (screening failure) may undergo rescreening. For rescreening, subjects still need to meet all participation criteria and sign the informed consent form.

- 1. **Lifestyle and Precautions**

During the study period, subjects should maintain a non-childbearing state and not be breastfeeding.

For premenopausal female subjects: Surgical sterilization can be used, or a medically recognized contraceptive method (such as intrauterine devices [IUD], oral contraceptives, or condoms) can be used during the study treatment period and for 6 months after the end of the study treatment.

Male subjects: Surgical sterilization can be used, or a medically recognized contraceptive method (such as condoms) can be used during the study treatment period and for 6 months after the end of the study treatment.

1. **Study treatment**
   1. **Study Medication and Administration**

The investigational drug, RC48 for injection, is provided free of charge by RemeGen, Ltd.

| Generic Name | RC48 |
| --- | --- |
| Trade Name | AidiXi |
| Company Code | RC48-ADC |
| Specification | 60mg/vial |
| Shelf Life | 24 months |
| Storage Conditions | Store and transport at 2℃～8℃, protected from light and sealed |
| Drug Characteristics | RC48 is a white to pale yellow fluffy substance. After reconstitution, it should be a colorless to pale yellow clear liquid. |
| Dosage | Dose Escalation. It is anticipated that three dose groups will be evaluated during the study period: 60mg, 120mg, and 180mg. |
| Preparation Method | 1. RC48 is a lyophilized powder preparation that requires reconstitution and dilution before intravesical administration. 2. Reconstitution Method: Each vial of RC48 contains 60mg of lyophilized powder, reconstituted with 6mL of sterile or bactericidal injectable water to form a 10mg/mL reconstitution solution. 3. Dilution Method: Dilute the reconstitution solution with 0.9% sodium chloride injection to a total volume of approximately 50mL. |
| Administration Method | The recommended procedure for intravesical RC48 is as follows and can be adjusted according to the clinical routine of the research center.   1. Within 2 hours before and until the completion of instillation, no large amounts of water should be consumed (not exceeding 50ml), no infusions should be administered, and no diuretic drugs should be taken. Instruct the subject to empty their bladder before instillation. 2. Strictly aseptic procedures should be followed for bedside catheterization. After draining residual urine and clamping the catheter, disinfect the instillation catheter interface; slowly inject the prepared medication into the bladder through the catheter, observing the subject's reaction during administration. If intolerable pain occurs, immediately stop the injection, rest and wait for improvement before continuing; for those who do not relieve after rest or have recurrent intolerable pain, cancel the instillation treatment. 3. Remove the catheter after injection. 4. Instruct the subject to avoid urination within 1 hour. To ensure full contact between RC48 and the bladder walls and achieve the therapeutic purpose, instruct the subject to roll over as much as possible, including supine, right lateral, prone, and left lateral positions, with each position lasting no less than 15 minutes. 5. Instruct the patient to urinate voluntarily after 1 hour and 10 minutes, with the maximum bladder retention time not exceeding 2 hours. If the retention time reaches 2 hours, suggest the patient urinate voluntarily; if unable to urinate, perform catheterization. 6. During or after bladder instillation or retention, if a hypersensitivity reaction occurs, slow down or interrupt the injection or instruct the patient to urinate, and provide appropriate medical treatment if necessary. For life-threatening drug-related reactions, terminate the medication immediately. |
| Dosing Schedule | 1. Start around 2-3 weeks post-surgery. Once a week for 6 consecutive weeks. 2. For patients receiving treatment, if there is no persistent/recurrent NMIBC, disease progression, or intolerable toxicity, they can obtain RC48 for maintenance treatment free of charge for up to one year. The medication plan for maintenance treatment is once every 4 weeks for a total of 11 times. Based on the individual response and tolerability of the subjects, open-dose-level maintenance instillation treatment is adopted, and dose escalation can be considered (see Appendix 5 Dose Escalation During Maintenance Instillation). |

The preparation and dilution of RC48 should refer to the “Instructions for Use of RC48”.

- 1. **Dose Adjustment of Study Medication**
     1. **Dose Adjustment During Dose Escalation Period**
        1. **DLT Evaluation Period (1-28 days)**

Dose adjustments are not allowed during the DLT evaluation period.

- - - 1. **Post-DLT Evaluation Period Treatment**

1. After completing the DLT evaluation period, if a subject experiences drug-related toxicity, first do not terminate the medication. Continue treatment with dose delay and reduction to maximize benefit while ensuring safety. For adverse reactions that are primarily systemic, consider dose reduction or deferral of intravesical RC48. If no relief is achieved, consider discontinuation of the medication. The reaction to drug-related toxicity should be judged by the investigator and corresponding treatment should be given.
2. Dose adjustments are decided by the investigator based on the subject's clinical condition, reducing the dose in increments of 30mg, and 30mg being the minimum dose.
3. Any reduction in dose for any reason is allowed up to 3 times. To avoid subjects being exposed to a lower dose that may not be therapeutically beneficial, if a fourth dose reduction is needed, the subject should be withdrawn from the study.
4. Subjects who have had a dose reduction and whose toxicity reactions have recovered are not allowed to increase the dose, to ensure good tolerability at the planned dose level.
5. If a subject experiences a drug-related adverse event, the medication can be postponed for up to 14 days. If the adverse event has not been relieved or recovered, it is recommended to terminate the treatment.
6. For subjects whose medication is postponed, the tumor assessment time points remain unchanged.

In general, when significant clinical significance of drug-related toxicity recovers to grade 1 or baseline, the subject can continue to use the study drug. During the recovery period, toxicity should be evaluated at least once a week. According to the investigator's judgment, the subject's continued administration can maintain the dose unchanged or reduce the dose, and the administration interval should continue as specified in the treatment plan.

- 1. **Overdose of Study Medication**

During the study, medication should be administered according to the dose and interval specified in the protocol, and overdose is not allowed.

If overdose of the test medication occurs, the amount used should be recorded in detail. Overdose is not recorded as an adverse event unless the investigator considers the adverse event to be related. If it meets the criteria for SAE, it should be recorded as a serious adverse event.

- 1. **Drug Interactions**
     1. **Drugs of the same class as anti-HER2 treatment**

No formal drug-drug interaction studies with RC48 have been conducted in patients.

- - 1. **The impact of other drugs on RC48**

CYP3A4 strong inhibitors: Other ADC drugs conjugated with MMAE, when used in combination with ketoconazole (a strong CYP3A4 inhibitor), will increase the exposure of free MMAE. It will cause C_max_ increase by 25%, AUC increase by 34% and no impact on ADC exposure. It is speculated that the impact of RC48 combined with strong CYP3A4 inhibitors on the exposure of free MMAE and conjugated antibody is the same as that of the ADC drug.

CYP3A4 strong inducers: Other ADC drugs conjugated with MMAE, when used in combination with rifampicin (a strong CYP3A4 inducer), will decrease the exposure of free MMAE. It will cause C_max_ decrease by 44%, AUC decrease by 46%, and no impact on ADC exposure. It is speculated that the impact of RC48 combined with strong CYP3A4 inducers on the exposure of free MMAE and conjugated antibody is the same as that of the ADC drug.

- - 1. **The impact of RC48 on other drugs**

CYP3A4 substrates: Other ADC drugs conjugated with MMAE, when used in combination with midazolam (a sensitive CYP3A4 substrate), do not affect the exposure of midazolam. It is speculated that RC48 will not affect the exposure of drugs metabolized by the CYP3A4 enzyme.

- 1. **Concurrent medications and treatments**

From 28 days before the first dose to 28 days after the last dose of the study medication, all concurrent medications (including permitted and not permitted) and treatments should be recorded in the original medical records and eCRF, and should be detailed in the original medical records and eCRF, including the name of the medication/treatment, reason for use, dose, unit, frequency, route, start date, and end date. Non-pharmacological treatments should include non-drug treatment name, treatment description, reason for treatment, start date, and end date.

- - 1. **Permitted concurrent medications and treatments**

During the study period, the investigator is allowed to carry out corresponding symptomatic treatment (medication) or supportive treatment (medication) according to the disease conditions of the subject. All concurrent medications/treatments need to be recorded, including prescription drugs, over-the-counter drugs, traditional Chinese medicine, etc. If there are changes in dose, frequency, etc., during the use of concurrent medications/treatments, these changes should be recorded.

All medications/treatments from 28 days before the first dose to 28 days after the last dose should be recorded.

- - 1. **Prohibited concurrent medications and treatments**

During the screening and treatment phases of the trial, subjects are prohibited from receiving the following treatments:

1. Systemic antitumor chemotherapy, radiotherapy, targeted, or biological treatment (except as specified in the protocol).
2. The protocol does not recommend the use of traditional Chinese medicine (TCM).
   - 1. **Emergency treatment of subjects**

Rapid systemic reactions

Clinical use of rapid systemic reactions is similar to infusion reactions, which often occur within the first hour of the first intravenous infusion, mainly manifested as fever, chills, difficulty breathing, bronchospasm, and other symptoms. Allergic reactions may also occur, such as hypotension and rash. Once the above adverse reactions occur during the bladder instillation or bladder retention process of RC48, the investigator should determine the severity and whether to stop the medication. Treatment methods include oxygen administration, conventional antipyretic analgesics (such as acetaminophen, etc.), antihistamines (such as diphenhydramine, etc.), corticosteroids, etc.

Bladder local reactions

If a subject experiences local bladder reactions after bladder instillation during the treatment, the investigator should assess and handle them according to NCI-CTCAE (version 5.0) and record them as AE.

- 1. **Supply, Packaging, Labeling, and Storage of Study Medication**

The medication supplier will provide enough study medication for the entire study period. These study medications will be packaged according to the study requirements, provided to the research center by the medication supplier, and received by authorized personnel involved in the study. The research center will store all study medications according to the requirements.

- 1. **Receipt, Counting, Distribution, and Storage of Study Medication**

The medication supplier will provide a sufficient amount of study medication to ensure the completion of the study. The study medication will be placed in a restricted access area and stored under conditions of light protection, 2-8℃.

The investigator is responsible for maintaining an accurate count record of the study medication during the clinical study period. The administration of the study medication to each subject will be recorded in the original medical records and eCRF.

1. **Screening and Treatment Period Assessments**
   1. **Demographic Characteristics**

Demographic characteristics include gender, date of birth, and ethnicity.

- 1. **Weight and Height**

Height is only recorded during the screening visit, and weight is measured at screening and before each administration of RC48.

- 1. **Vital Signs and Physical Examination**

Vital signs include blood pressure, respiration, pulse, and body temperature.

Physical examination includes skin, mucous membranes, lymph nodes, head and neck, chest, abdomen, spine/extremities, nervous system, and others.

- 1. **Medical History and Other Past Histories**

Medical history includes a history of non-muscle-invasive bladder cancer (NMIBC), other tumor histories, and non-tumor histories. Non-tumor medical history refers to clinically significant past diseases and concomitant diseases (or signs and symptoms, if a diagnosis cannot be determined) that occurred before the study medication is given. Additionally, a history of alcohol abuse, drug abuse, allergy, and surgery will also be recorded as other past histories in the original medical records and eCRF.

- 1. **History of Non-Muscle-Invasive Bladder Cancer (NMIBC)**

During the screening period, the following information about the subject's study disease history should be recorded:

1. The date of the first diagnosis of NMIBC, the location of the primary site, the pathological histological type and grade (1973 and 2004 WHO grading systems), tumor staging (TNM staging), risk group classification, see Appendix 14 of the protocol for details.
2. The number of previous recurrences (if any).
3. The lesion site of the TURBT procedure within 3 weeks before the start of treatment, the pathological results of the tumor tissue obtained from this TURBT (pathological histological type and grade, whether it invades the muscle layer, whether it invades the lamina propria of the mucosa, whether there is vascular and lymphatic invasion, whether there is a pathological subtype of urothelial carcinoma (such as papillary type, etc.), and whether there is carcinoma in situ (CIS) and other prognostic information), tumor staging (TNM staging), and confirm the diagnosis of high-risk NMIBC, the pathological grading of NMIBC, and tumor staging.
4. The treatment history of NMIBC, including surgical history (surgical method, date of surgery), drug treatment (such as bladder instillation, systemic treatment), radiotherapy, etc., especially the drug treatment history immediately after TURBT within 3 weeks before the start of treatment.
5. Additionally, the two types of populations in the study should record the following information:

- High-risk NMIBC patients who have not previously received BCG treatment: The subject's history of not receiving BCG treatment and not meeting or refusing radical cystectomy.
- High-risk NMIBC patients who are unresponsive to BCG: The subject's history of BCG treatment, the persistence/recurrence of the disease after BCG treatment, and not meeting or refusing radical cystectomy.
  1. **HER2 (IHC) Testing of Tumor Tissue**

The HER2 (IHC) testing and result reporting of the NMIBC tumor pathological tissue specimens obtained from the TURBT procedure within 3 weeks before the first administration of the study medication are conducted by the research center.

- 1. **Previous Medication/Concurrent Medication and Treatment**

Previous medication/treatment refers to non-antitumor drugs/treatments used within 28 days before the first administration of the study medication, including prescription drugs, over-the-counter drugs such as aspirin, folic acid, vitamins, minerals, dietary supplements, etc.

Concurrent medication/treatment refers to two or more drugs/treatments administered simultaneously. In the study, it is specified as any drug/treatment administered at the same time/period from the first dose of the study medication until the completion of the study safety visit.

From the screening period to 28 days after the last study medication, the investigator should inquire about any medication/treatment used by the subject since the last visit. Any previous medication and concurrent treatment will be recorded in the original medical records and eCRF, including all records of previous medication and concurrent medication: drug/treatment name, reason for use, dose, unit, frequency, route, start date, and end date; previous/concurrent treatment should include: non-drug treatment name, treatment description, reason for treatment, start date, and end date. If the reason for concurrent medication and treatment meets the definition of AE, the relevant information should also be recorded in the subject's original medical records and eCRF.

- 1. **Electrocardiogram (ECG)**

A 12-lead electrocardiogram is conducted by the research center.

- 1. **Echocardiogram**

An echocardiogram is conducted by the research center.

- 1. **Laboratory Tests**

Laboratory tests (complete blood count, blood biochemistry, urine routine, and coagulation function) are conducted by the research center.

- 1. **Cytology Examination (Urine Cytology)**

Urine cytology examination is conducted, and the results of specimens collected from natural urination, catheterization, and bladder washing during cystoscopy are accepted.

- 1. **Pregnancy Test**

Women of childbearing age will undergo a pregnancy test to exclude pregnancy, and only blood pregnancy test results are accepted.

1. **Safety Assessments**
   1. **Definitions**
      1. **Adverse Event (AE)**

AEs refer to all adverse medical events that occur in subjects after receiving the test medication, which can manifest as symptoms, signs, diseases, or laboratory abnormalities but are not necessarily related to the test medication.

- - 1. **Adverse Drug Reaction (ADR)**

Adverse drug reactions refer to any harmful or unexpected reactions related to the test medication that occur during a clinical trial. There must be at least a reasonable possibility that the test medication is associated with the adverse event, i.e., the relationship cannot be ruled out.

- - 1. **Drug-related Adverse Event (DRAE)**

AEs that occur after the start of treatment with the test medication or after the exacerbation of preexisting medical conditions before treatment.

- - 1. **Severe Adverse Event (SAEs)**

SAEs are those that meet any of the following criteria: resulting in death; life-threatening; requiring hospitalization or prolonged hospitalization; resulting in permanent or significant disability or loss of function; or congenital anomalies or birth defects, leading to other significant medical events that may not immediately threaten life, death, or hospitalization but require medical intervention to prevent one of the above situations. See the table below for details:

| Serious Adverse Event | Definition |
| --- | --- |
| Death | Must fully document and report any event that results in a fatal outcome, regardless of whether the event is related to the study medication. The death of a subject in the trial itself is not an adverse event but the result of an adverse event. |
| Life-threatening | Life-threatening refers to the direct risk of death faced by the subject when the event occurs, not an event that could potentially lead to death if the situation worsens. |
| Hospitalization or prolonged hospitalization | Refers to hospitalization or prolongation of hospital stay due to adverse events (excluding social reasons such as medical insurance reimbursement, medical procedures, and research procedures). The following hospitalizations are not considered SAEs:   1. Planned hospitalizations (e.g., elective or scheduled surgeries arranged before the start of the study; hospitalization is part of the research procedure). 2. Hospitalizations not related to adverse events (e.g., hospitalizations for short-term care purposes). |
| Permanent or significant disability or loss of function | Disability/loss of function refers to a severe impairment in the subject's ability to carry out normal life activities. If in doubt, the investigator should make a medical judgment. |
| Congenital anomalies/birth defects | Refers to congenital anomalies/birth defects observed in offspring conceived during the subject's exposure to the study medication. |
| Significant medical events /other | Must use medical and scientific judgment to decide whether to expedite reporting of other situations, such as significant medical events that may not immediately threaten life, death, or hospitalization, but require medical intervention to prevent one of the above situations. For example, significant treatment in the emergency room or at home for allergic bronchospasm, unhospitalized cachexia or convulsions, drug dependence or addiction, etc. |

- 1. **Collection and Recording of Safety Information**

The investigator is responsible for collecting all adverse medical events from the time the subject signs the informed consent form until the last treatment within the study period (excluding the maintenance instillation period) ends 28 days. Adverse medical events occurring after the subject signs the informed consent form but before the first use of the study medication should be recorded as medical history/concomitant diseases in the CRF. However, if they meet any of the following conditions, they should be handled and reported as adverse events/serious adverse events: any harm/damage caused by research procedures, adverse medical events caused by stop treatment related to the research protocol, or adverse medical events related to research procedures.

From the time the subject first uses the study medication until 28 days after the last study medication treatment, all adverse events should be collected and recorded. During the collection and evaluation of adverse events, it is necessary to clarify the name of the adverse event, determine the start and end times of the event, assess the severity of the event, the investigator's judgment of the correlation with the test medication, the measures taken for the medication, the outcome, etc., and describe the event.

After the last study medication treatment ends 28 days later, if the investigator learns of any adverse events and believes they are reasonably related to the study medication, they should be recorded and reported according to the adverse event handling procedures.

Sources of AEs generally include:

1. The investigator's inquiry about the occurrence of AEs and the subject's response regarding their health status. Each time the investigator asks open-ended questions (e.g., "Have you noticed any changes since our last meeting?" or "Do you have any other concerns?"), the investigator should avoid influencing the subject's answers with their own questioning method.
2. Symptoms spontaneously reported by the subject.
3. The investigator assesses that clinically significant changes or abnormalities found or detected are recorded as adverse events.
4. Other information related to the subject's health that the investigator becomes aware of.

The investigator must record each subject's adverse events on the electronic data capture system (EDC). This includes AEs that meet the reporting criteria for SAEs. AEs that meet the definition of SAEs should be reported according to the serious adverse event reporting procedures. The records mainly include:

1. Adverse Event Name

All AEs should be described using medical professional terminology as much as possible, recorded according to the exact medical diagnosis, and named with the name of the disease, condition, or syndrome, rather than the name of each individual sign or symptom. If a group of signs and/or symptoms cannot be medically classified as a diagnosis or syndrome when reported, each individual event should be recorded separately on the relevant forms (SAE report form and/or eCRF). If a confirmed diagnosis is subsequently obtained, the confirmed diagnostic name should be reported when follow-up information is obtained. If a group of symptoms is reported and a diagnosis is later confirmed, the signs/symptoms should be updated to reflect the content of the diagnosis.

When determining the name of the adverse event, it should be ensured that each adverse event name consists of a single event, and a diagnosis, symptom/sign is an adverse event.

Hospitalization, surgery, death, and other terms are not adverse events themselves, and the causes leading to the above conditions need to be recorded as adverse events. When the cause of the above conditions is not yet determined, the known information, such as hospitalization, death, etc., can be used as the name of the adverse event, and the above information should be updated in subsequent follow-ups.

1. Adverse Event Start Time

The start time of an adverse event is the date when the first symptom or sign was first observed. If the adverse event is a clinically significant abnormal laboratory test or examination result, the start time is based on the sampling date.

For adverse events that progress to serious adverse events, the occurrence time of the serious adverse event starts from the date when the adverse event is upgraded to a serious adverse event.

1. Adverse Event End Time

The end time of an adverse event should be the time when the adverse event is cured, improved, or ends. The time should be as precise as possible to the year, month, and day. If the information is incomplete, it should also be specific to the year and month.

If the subject dies and the end time of the adverse event that is not the direct cause of death is not collected, the end time of the adverse event that still persists should be left blank, and the status should be "ongoing". If it is judged to be the direct or main cause of "death", the end time of the adverse event is the time of death.

If the duration of the adverse event is within 24 hours, the duration should be recorded in hours.

1. Adverse Event Severity

The severity of adverse events should be recorded using the National Cancer Institute's Common Terminology Criteria for Adverse Events (NCI-CTCAE) version 5.0. If the severity of individual adverse events is not graded in the guidance document, the investigator can use the general definition of grades 1 to 5 for grading based on their medical judgment:

- Grade 1: Mild; asymptomatic or mild; only seen clinically or diagnostically; no treatment required.
- Grade 2: Moderate; requires minor, local, or non-invasive treatment; limitations in instrumental activities of daily living*.
- Grade 3: Severe or medically significant but not immediately life-threatening; hospitalization or prolongation of hospital stay; disability; limitations in self-care activities of daily living**.
- Grade 4: Life-threatening; requires urgent intervention.
- Grade 5: Death related to the AE.

*Instrumental activities of daily living refer to cooking, shopping for clothes, using the phone, managing finances, etc.

**Self-care activities of daily living refer to bathing, dressing, eating, washing, taking medication, etc., and not bedridden.

1. Measures Taken for the Study Medication Due to Adverse Events

Measures taken for the study medication due to adverse events are as follows: dose unchanged, dose reduction, hold medication, discontinue medication, not applicable.

If necessary, timely treatment should be given to protect the health and interests of the subject. Emergency treatment should be provided in case of emergency. If the study medication is used to treat adverse events, it should be recorded in the concomitant medication record.

1. Evaluation of Causal Relationship between Adverse Events and Test Medication

The correlation between adverse events and the study medication in this study uses the following five standards: "Definitely related", "Highly likely", "Possible", "Unlikely", "Unrelated". The details are as follows:

| Unrelated | The adverse event is caused by other factors, such as the subject's clinical condition, other treatments, or concomitant medications. |
| --- | --- |
| Possible unrelated | The occurrence of the adverse event may be caused by other factors, such as the subject's clinical condition, other treatments, or concomitant medications, and is inconsistent with the known information about the study medication. |
| Possible | The adverse event is consistent with the known information about the study medication and has a causal relationship with the study medication, but it may also be related to other factors. |
| Highly likely | The adverse event is consistent with the known information about the study medication and has a causal relationship with the study medication, and cannot be explained by other factors, such as the subject's clinical condition, other treatments, or concomitant medications. |
| Definitely related | The adverse event is consistent with the known information about the study medication and has a causal relationship with the study medication, and this relationship cannot be explained by other factors, such as the subject's clinical condition, other treatments, or concomitant medications. In addition, the adverse event reoccurs when the subject takes the study medication again. |

If judged as definitely related, highly likely, and possible, it should be considered as treatment-related adverse events (TRAEs) or adverse drug reactions.

7. Outcome of Adverse Events

The outcomes of adverse events can have the following statuses: resolved, improved/relieved, not improved/unrelieved/ongoing, resolved with sequelae, fatal, unknown.

- 1. **Adverse Event Assessment**

Significant abnormalities in vital signs and physical examination results compared to baseline will be reported as adverse events.

The investigator will review all laboratory test results, evaluate the clinical significance of the subject's laboratory test results relative to baseline based on the laboratory reference range, determine the clinical significance of each abnormal test value, and record them as "abnormal but not clinically significant (NCS)" or "abnormal and clinically significant (CS)". Abnormal laboratory tests that occur during the study, if they meet any of the following conditions, should be judged as adverse events and recorded in the original medical records and eCRF:

1. This abnormality indicates a disease and/or new, or worsening organ toxicity compared to baseline.
2. This abnormality requires adjustment of the dose or method of administration of the test medication, such as changes in drug dosage, discontinuation of medication, etc.
3. This abnormality requires additional active intervention, such as increased or changed concomitant medication, close observation, more frequent follow-up assessments, and further diagnosis.

If unexplained abnormalities in laboratory test values occur, retesting and follow-up should be conducted until they return to normal range or baseline, and/or a reasonable explanation for the abnormal test values is found. If a clear explanation is obtained, it should also be recorded on the eCRF.

New primary tumors refer to tumors that are not the main target of the study medication treatment and occur after the patient is included in the study. New primary tumors should be judged as AEs, but do not include metastatic foci of the original tumor. Metastatic foci symptoms or metastatic foci themselves should not be reported as AEs/SAEs, as they will be considered disease progression. During the study, symptoms and signs that deteriorate in severity or frequency due to changes or unexpected progression of the disease nature, as judged by the investigator, should be reported as adverse events.

Definite tumor progression symptoms or signs should not be recorded as adverse events unless the investigator judges them to be more severe than expected or considers the tumor progression to be related to the test medication or research procedures. If an event cannot be determined to be caused by disease progression, it should be recorded as an adverse event.

- 1. **Adverse Event Assessment and Follow-up Time Limits and Frequency**

During the study period, the investigator must follow up on each adverse event/serious adverse event until it is resolved, stable, reasonably explained, returned to baseline, the subject is lost to follow-up, or the subject dies.

- 1. **Serious Adverse Event Reporting**

Any serious adverse event that occurs during the clinical trial, regardless of whether it is related to the treatment, should be immediately reported by phone to the principal investigator (PI) and the ethics committee within 24 hours. At the same time, the investigator must fill out the "Post-Marketing Adverse Drug Reaction Report Form", record all the information involved in the report (if collectible), and report it to the RemeGen, Ltd. Pharmacovigilance Department within 24 hours.

If the investigator cannot promptly learn of serious adverse events (such as when the subject first seeks care at an external hospital), but should report and record the time they first learn of the serious adverse event within 24 hours.

- 1. **Pregnancy Events**

The investigator will collect pregnancy events confirmed for subjects (or their partners) from the start of the first study treatment until 6 months after the last study treatment. If the investigator suspects pregnancy after the administration of the study medication, the study medication must be immediately discontinued until the pregnancy outcome is confirmed. Once a pregnancy event occurs during the trial, the investigator should communicate with the subject in a scientific and rigorous manner, informing her/him of the potential effects and risks of the test medication for pregnant women and fetuses. If a female subject experiences a pregnancy event, the investigator should immediately terminate the study treatment.

After confirming a pregnancy event for a subject or their partner, the investigator should record and report it. In addition, the investigator should follow up the pregnancy until it ends or is terminated (such as: induced abortion, spontaneous abortion, stillbirth, neonatal death, spontaneous abortion, medically indicated termination of pregnancy, etc.). If a subject or their partner's pregnancy leads to an abnormal pregnancy outcome (such as: congenital malformations, birth defects, stillbirth, neonatal death, spontaneous abortion, termination of pregnancy for medical reasons, etc.), the investigator should report it according to the SAE reporting procedure.

1. **Study Schedule**
   1. **Study Visit Assessment Schedule**

|  | Screening Period | Treatment Period | End of Treatment | |
| --- | --- | --- | --- | --- |
| Visit | -28 to -1 day | 1 to 42 days | Discontinu-ation of treatment ±7 days | 28 days ±3 days after the last dose |
| Informed Consent ^a^ | × |  |  |  |
| Inclusion/Exclusion Criteria | × |  |  |  |
| Demographic Information | × |  |  |  |
| Medical History and Other Past Histories ^b^ | × |  |  |  |
| NMIBC History | × |  |  |  |
| Tumor Tissue HER2 (IHC) Test ^c^ | × |  |  |  |
| Height | × |  |  |  |
| Weight ^d^ | × | × |  |  |
| Vital Signs ^e^ | × | × | × |  |
| Physical Examination ^f^ | × | × | × |  |
| Complete Blood Count ^g^ | × | × | × |  |
| Blood Biochemistry ^h^ | × | × | × |  |
| Coagulation Function Test ^i^ | × |  | × |  |
| Urine Routine ^j^ | × | × | × |  |
| Serum Virology Test ^k^ | × |  |  |  |
| Electrocardiogram (ECG) ^l^ | × | × | × |  |
| Echocardiogram ^m^ | × |  | × |  |
| Chest PA X-ray or Chest CT Scan ^n^ | × |  |  |  |
| Pregnancy Test ^o^ | × |  | × |  |
| ECOG Performance Status ^p^ | × |  | × |  |
| Intravesical RC48 ^q^ |  | × |  |  |
| Adverse Events/Concomitant Medications | × | × | | |

^a^ Informed consent should be obtained before all screening procedures.

^b^ Medical history refers to clinically significant past and concomitant disease information (or signs and symptoms, if a diagnosis cannot be determined) that occurred before the study medication is given. Additionally, a history of alcohol abuse, drug abuse, allergy, and surgery will also be recorded as past medical history in the original medical records and eCRF.

^c^ Tumor tissue HER2 (IHC) testing is conducted during the screening period, and NMIBC tumor pathological tissue specimens obtained from TURBT surgery within 3 weeks before the first dose are tested for HER2 (IHC) and reported by each research center.

^d^ Weight is measured before each administration of RC48.

^e^ Vital signs include blood pressure, respiration, pulse, and body temperature, measured before each administration.

^f^ Physical examination includes skin, mucous membranes, lymph nodes, head and neck, chest, abdomen, spine/extremities, nervous system, and others, conducted during the screening period, within 3 days before the fourth week of treatment, and at the exit visit.

^g^ Complete blood count includes red blood cell count (RBC), hemoglobin concentration (HGB), hematocrit (HCT), eosinophil count, basophil count, white blood cell count (WBC), platelet count (PLT), absolute neutrophil count (ANC), and absolute lymphocyte count (ALC). If the test within the screening period is completed within 7 days before the first dose, the test before the first dose does not need to be repeated. It is recommended to perform the test within 3 days before each administration of RC48 and at the exit visit.

^h^ Blood biochemistry includes alanine aminotransferase (ALT), aspartate aminotransferase (AST), total bilirubin (TBIL), alkaline phosphatase (ALP), lactate dehydrogenase (LDH), gamma-glutamyltransferase (GGT), total protein (TP), albumin (ALB), total cholesterol (TC), triglycerides (TG), urea (UREA) or blood urea nitrogen (BUN), creatinine (Cr), glucose (GLU), potassium (K^+^), sodium (Na^+^), chloride (Cl^-^), calcium (Ca^2+^), and phosphorus (P). If the test within the screening period is completed within 7 days before the first dose, the test before the first dose does not need to be repeated. RC48 is administered within 3 days before each dose and at the exit visit.

^i^ Coagulation function includes prothrombin time (PT), activated partial thromboplastin time (APTT), thrombin time (TT), and fibrinogen (FBG), conducted during the screening period and at the exit visit.

^j^ Urine routine includes urine glucose, urine protein, urine red blood cells, urine white blood cells, and epithelial cells. If the test within the screening period is completed within 7 days before the first dose, the test before the first dose does not need to be repeated. RC48 is administered within 3 days before each dose and at the exit visit.

^k^ Serum virology tests include hepatitis B panel, hepatitis C antibody (HCVAb), and human immunodeficiency virus antibody (HIVAb). If hepatitis B surface antigen is positive, further testing for HBV DNA quantification is required. If hepatitis C antibody is positive, further testing for HCV RNA quantification is required. Available test results within 6 months before enrollment are acceptable.

^l^ 12-lead electrocardiogram is conducted during the screening period, within 3 days before the fourth week of treatment, and at the exit visit. If the test within the screening period is completed within 7 days before the first dose, the test before the first dose does not need to be repeated.

^m^ Echocardiogram is conducted during the screening period and at the exit visit.

^n^ Chest PA X-ray or chest CT scan is only conducted during the screening period. Available test results within 3 months before enrollment are acceptable.

^o^ Pregnant women of childbearing age will undergo a pregnancy test to exclude pregnancy, and only blood pregnancy test results within 7 days before the first dose are accepted. Suspected subjects during the study and at the exit visit will undergo blood pregnancy testing again.

^p^ ECOG performance status is conducted during the screening period, within 3 days before the fourth week of treatment, and at the exit visit.

^q^ Intravesical RC48 is given once a week for 6 consecutive weeks, with a window of ±1 day, followed by a 4-week break. For patients receiving treatment, if there is no persistent/recurrent NMIBC, disease progression, or intolerable toxicity, they can obtain RC48 for maintenance treatment free of charge for up to one year. The medication plan for maintenance instillation treatment is once every 4 weeks for a total of 11 times.

- - 1. **Screening Period (Days -28 to -1)**

1. All subjects must sign the Informed Consent Form before initiating the screening procedures.
2. Collect demographic characteristics, weight, height, medical history and other past histories, tumor history.
3. Collect information on previous medication/treatment within 28 days prior to the first study drug administration.
4. Conduct vital signs and physical examinations.
5. Perform routine blood tests, blood biochemistry, urine routine, coagulation function tests, 12-lead ECG, and echocardiogram (results from tests conducted by the research center are acceptable, but must be completed within 28 days before the start of the first study drug administration), pregnancy test (blood pregnancy test within 7 days before the first dose).
6. Conduct serum virology tests: Hepatitis B panel, Hepatitis B virus DNA quantification (when HBsAg is positive), Hepatitis C antibody (HCVAb), Hepatitis C HCVRNA quantification (when HCVAb is positive), Human Immunodeficiency Virus antibody (HIVAb), results from within 6 months before the start of the first study drug administration are acceptable.
7. Obtain TURBT surgery reports and biopsy pathology results within 3 weeks before the first dose; based on the clinical judgment of the investigator and the specific circumstances of the patient, consider performing urine cytology examination and urological CT scan with contrast enhancement + urography (CTU) (if performed, results from within 3 months before the first dose are acceptable).
8. HER2 (IHC) testing of NMIBC tumor tissue obtained from TURBT surgery within 3 weeks before the first dose is conducted by the research center.
9. Chest PA X-ray or chest CT scan (results from within 3 months before the first dose are acceptable).
10. ECOG performance status.
11. Confirm that all inclusion criteria are met and none of the exclusion criteria are met.
12. Collect adverse events.
    - 1. **Treatment Period**
         1. **Study Medication Administration**

Intravesical RC48 is given once a week for 6 consecutive weeks, with a window of ±1 day, followed by a 4-week break. For patients receiving treatment, if there is no persistent/recurrent NMIBC, disease progression, or intolerable toxicity, they can obtain RC48 for maintenance treatment free of charge for up to one year. The medication plan for maintenance instillation treatment is once every 4 weeks for a total of 11 times. Based on the individual response and tolerability of the subjects, open-dose-level maintenance instillation treatment is adopted, and dose escalation can be considered (see Appendix 5 Dose Escalation During Maintenance Instillation).

- - - 1. **Treatment Period Study Schedule**

1. Weight measurement before each administration.
2. Vital signs measurement before each administration.
3. Within 7 days before the first administration, conduct vital signs, physical examination, routine blood tests, blood biochemistry, and urine routine tests. If the screening period tests are completed within 7 days before the first administration, routine blood tests, blood biochemistry, and urine routine tests do not need to be repeated.
4. Except for the first administration, RC48 requires vital signs, routine blood tests, blood biochemistry, and urine routine tests within 3 days before each administration.
5. Collect information on concomitant medication/treatment and adverse events.
   - - 1. **Exit Visit (±7 days after the last dose)**
6. Conduct vital signs and physical examination.
7. Perform routine blood tests, blood biochemistry, urine routine, coagulation function tests, 12-lead ECG, echocardiogram (if coincident with other visits, no repetition is necessary).
8. ECOG performance status.
9. Pregnancy test (blood pregnancy test).
10. Collect information on concomitant medication/treatment and adverse events.
    - - 1. **Safety Visit**

Conducted 28±3 days after the last treatment, collect information on concomitant medication/treatment and adverse events within 28 days after the last dose.

- - 1. **Unscheduled Visits**

Any unscheduled visits during the study period must be recorded in the original medical records and eCRF. During unscheduled visits, record relevant examination content based on the purpose of the visit (such as: vital signs, physical examination, adverse events, concomitant medication/treatment, etc., as deemed necessary by the investigator).

1. **Statistical Analysis**
   1. **Statistical Analysis Plan**

For detailed statistical analysis, refer to the "Statistical Analysis Plan (SAP)." Before database lock, statisticians will discuss with the principal investigators and RemeGen, Ltd. to finalize the plan based on data characteristics.

- 1. **Sample Size Estimation**

It is preliminarily estimated that approximately 9 subjects are needed to determine the MTD (Maximum Tolerated Dose). Subjects with unassessable DLTs (Dose-Limiting Toxicities) will be supplemented. The actual number of subjects receiving treatment will depend on the occurrence of adverse reactions during the study period, which may result in more subjects being enrolled than initially anticipated.

- 1. **Analysis Populations**

The statistical analysis populations and result variables are summarized as follows:

| Result Parameters | Analysis Populations |
| --- | --- |
| DLT incidence, MTD, RP2D | DLT Analysis Set |
| 3-month, 6-month, 12-month DFS (Disease-Free Survival) rates | FAS (Full Analysis Set) |
| RFS (Recurrence-Free Survival), PFS (Progression-Free Survival), OS (Overall Survival) | FAS |
| Complete Response (CR) rate | Subgroup population with CIS at baseline |
| Baseline characteristics | SS (Safety Set) |
| Study drug exposure | SS |
| Adverse events | SS |
| Laboratory tests | SS |
| Vital signs and physical examinations | SS |

- - 1. **Full Analysis Set (FAS)**

The collective set of all subjects who have been enrolled and have taken the study drug at least once.

- - 1. **DLT Analysis Set**

The DLT Analysis Set includes subjects who have completed at least one study drug treatment during the dose-escalation phase, completed the DLT assessment observation period (Day 1 to Day 28 observation period), and received adequate safety assessments, or experienced a DLT. The DLT Analysis Set is only applicable to Phase I studies.

- - 1. **Safety Analysis Set (SS)**

The Safety Analysis Set (SS) consists of subjects who have received at least one dose of the study drug and have safety evaluations.

- 1. **Statistical Analysis Methods**
     1. **General Considerations**

SAS 9.4 (or higher version) software will be used for analysis. Unless otherwise specified, descriptive statistics for continuous variables include the number of cases, missing numbers, mean, standard deviation, median, quartiles, minimum, and maximum. Unless otherwise specified, descriptive statistics for categorical variables are frequencies, missing numbers, and percentages. The denominator for percentage calculations is the number of subjects in each group of the analysis set.

This study is exploratory in nature and does not conduct confirmatory statistical hypothesis testing.

- - 1. **Demographic Data and Basic Characteristics**

Analysis will be based on actual data in the SS set. Demographic data and other baseline characteristics will be descriptively statistically analyzed by dose group, including age, gender, ethnicity, height, weight, subject baseline disease characteristics, medical history, and treatment status, etc.

- - 1. **Evaluation Indicator Analysis**
       1. **Safety Analysis**

Summarize the occurrence of dose-limiting toxicities (DLT) in each dose group and conduct safety analysis.

List by dose group and primary system organ class (SOC)/preferred term (PT).

The maximum tolerated dose (MTD) and recommended phase II dose (RP2D) will be assessed by the investigators based on the occurrence of drug DLTs.

Adverse event occurrences are limited to treatment-emergent adverse events (TEAEs). TEAEs are defined as:

Adverse events occurring from the start of trial medication to the 28th day after the last dose, and adverse events related to the trial medication after the 28th day of the last dose, or exacerbations or worsening of pre-existing adverse events or medical conditions before medication use.

All adverse events will be graded according to NCI-CTCAE (version 5.0) and coded according to the current version of MedDRA at the time of initial coding, and processed in statistical analysis.

Statistics of TEAEs, adverse reactions (AEs with a definite, likely, or possible relationship to the trial medication are adverse reactions), serious adverse events, serious adverse reactions, adverse events or reactions with severity ≥ Grade 3, adverse events or reactions leading to withdrawal from the trial, adverse events leading to dose reduction, adverse events leading to drug suspension, adverse events leading to termination of treatment, and adverse events leading to death, etc., will be provided with the number of cases and percentage. Lists of TEAEs, adverse reactions, serious adverse events, adverse events leading to withdrawal from the trial, adverse events leading to termination of treatment, adverse events leading to death, and subjects who died during the treatment phase, and summarize the number and percentage of adverse events and adverse reactions by SOC and PT according to the severity of adverse events.

Provide data lists for abnormal results and clinical descriptions of vital signs, physical examinations, laboratory tests, and other examinations.

The occurrence of DLTs, MTD, and RP2D assessments will be based on the DLT Analysis Set, and other safety evaluations will be based on the Safety Analysis Set (SS). Quantitative data will be descriptively statistically analyzed for various examinations by dose group at baseline and post-dose at each scheduled visit (mainly including the number of cases, mean, standard deviation, median, minimum, and maximum), and provide a table of clinical significance relative to baseline changes at each visit post-dose. Qualitative data will provide a table of clinical significance relative to baseline changes at each visit post-dose by dose group.

1. **Data Collection and Management Responsibilities**

This study will use an electronic data capture (EDC) system, and study data will be entered into eCRFs by investigators or authorized study center staff.

eCRFs should be completed as soon as possible during or after visits and updated at any time to ensure they reflect the latest dynamics of subjects participating in the study. To avoid differences in result evaluation between different investigators, it should be ensured that the baseline and all subsequent efficacy and safety evaluations for the same subject are completed by the same person. Investigators must review the data to ensure the accuracy and correctness of all data entered into eCRFs. If some assessments are not performed during the study process, or some information is not available, not applicable, or unknown, investigators should record them truthfully in eCRFs. Investigators should electronically sign the data after verification.

Unless otherwise specified, eCRFs will only be used as forms for data collection and cannot serve as original data. Original documents refer to those used by investigators or study centers that are related to subjects and can prove the existence of subjects, inclusion and exclusion criteria, and all records of their participation in this study, including laboratory records, ECG results, subject folders, etc.

Investigators are responsible for maintaining all original documents and ensuring that clinical monitors (CRA) inspect them during each visit. In addition, regardless of the duration of the subject's participation in the study, investigators must submit complete eCRFs for each subject participating in the study. All supporting documents (such as laboratory records or study center records) submitted with eCRFs should be carefully verified for research numbers and subject numbers, and all personal privacy information (including subject names) should be deleted or made unrecognizable to protect subject privacy.

When study data is entered into eCRFs, the system will automatically record the entry trajectory of the data entrant through the login ID of the data entrant. Investigators record proof that they have reviewed the record and ensure the accuracy of the data through electronic signatures. Electronic signatures will be completed using the investigator's user ID and password, and the system will automatically attach the date and time of the electronic signature. If changes are needed in the eCRF data, they should be made according to the workflow defined by the EDC system. All changes and reasons for changes will be recorded in the audit trail.

CRA will review the original medical records and eCRFs, and assess their completeness and consistency, and CRA will compare eCRFs and original documents with other relevant documents to ensure the completeness and consistency of key data.

All data entry, corrections, and modifications will be the responsibility of the investigator or their authorized personnel, and monitors do not have this authority. Data submitted to the data server in eCRFs, any changes to the data will be recorded in the audit trail, that is, the reason for the change, the operator's name, modification time, and date will be recorded. The roles and permissions of the staff responsible for data entry at the study center will be predetermined. If there are data queries, CRA, data managers, or medical monitors will issue queries in EDC, and study center staff will be responsible for answering questions. The EDC system will record the audit trail of queries, including the investigator's name, time, and date.

- 1. **Study Record Retention**

During the implementation of the study and after the study is completed, the study center will archive necessary documents in accordance with the requirements of Chinese GCP.

In addition, these documents will be kept until:

1. At least 5 years after the study ends or is terminated early.
2. Or at least 5 years after the final approval of the Chinese marketing application, and until there are no pending or under review marketing applications in China.
3. Or at least 5 years after the formal termination of the clinical development project of the study drug; choose the longer of the two time periods.
4. **Supporting Documents and Operational Considerations**
   1. **Regulations, Ethics**
      1. **Informed Consent Process**
         1. **Informed Consent Form and Other Documents Provided to Subjects**

This clinical trial must follow the Declaration of Helsinki (2013 version), the Good Clinical Practice (GCP) for drug clinical trials issued by NMPA, and relevant regulations.

If issues arise in the actual implementation of the clinical study according to this plan, it is necessary to discuss and revise the plan by the responsible unit of the clinical study, and then implement it after re-approval by the ethics committee. If important new information involving the study drug is found, it is necessary to modify the informed consent form in writing and obtain the subject's consent again after approval by the ethics committee.

- - - 1. **Informed Consent Process and Documents**

Before the start of the subject's clinical study, the investigator must provide the subject with detailed information about the clinical study, including the nature of the study, the purpose of the study, possible benefits and risks, other available treatment methods, and the rights and obligations of the subject in accordance with the Declaration of Helsinki, so that the subject's legal representative can fully understand and consent to sign the informed consent form before starting the clinical study. Each patient must leave detailed contact information, phone number, ID card, etc., and the doctor must also leave their contact phone number for the patient, so that the patient can find the doctor at any time when there is a change in condition, which is also conducive to the doctor's timely understanding of the condition changes, reminding patients to return to the clinic in time, and avoiding missed visits.

- - 1. **Confidentiality and Privacy**

All clinical study results and documents must be kept confidential. Such information must not be disclosed without the consent of the investigator and members of their research team.

The identity of subjects participating in the study must not be disclosed. eCRFs and other documents can only represent subjects with subject numbers, initials, or dates of birth, and must not include the subject's name. Investigators must keep files that indicate the subject's identity (e.g., signed subject information and informed consent forms) confidential.

- - 1. **Quality Assurance and Quality Control**

This study will be monitored according to Chinese GCP.

This study requires that monitors can directly access original data for verification by comparing the data on the subject's eCRF with the data on their original documents. This data verification process is an important part of ensuring the quality of the study, and it is possible to correct transcription errors and omissions during this process.

- - 1. **Protocol Deviation**

During the study, medication should be administered according to the dosage and interval specified in the protocol. It is not allowed to administer medication early or in excess.

If the protocol specifies the time for vital sign observations, they should be conducted as close as possible to the specified time, but exceeding the specified time range will not affect the evaluation results. Therefore, data on vital sign observations do not determine whether a subject is excluded from the evaluation.

Laboratory examination information should be complete; incomplete information during the screening period will not be admitted to the study. It should be completed according to the protocol during the study period. At least, follow-ups for abnormalities with clinical significance should be tracked until they return to normal or stabilize or baseline. After the last visit, if the subject is unwilling to return, their condition should be inquired about in detail and recorded. If the subject does not answer the phone, the time and number of calls should also be recorded.

1. **References**

1. Antoni S, Ferlay J, Soerjomataram I, Znaor A, Jemal A, Bray F. Bladder Cancer Incidence and Mortality: A Global Overview and Recent Trends. *Eur Urol* 2017; **71**(1): 96-108.

2. Woldu SL, Bagrodia A, Lotan Y. Guideline of guidelines: non-muscle-invasive bladder cancer. *BJU international* 2017; **119**(3): 371-80.

3. Hussain MH, Wood DP, Bajorin DF, et al. Bladder cancer: narrowing the gap between evidence and practice. *Journal of clinical oncology : official journal of the American Society of Clinical Oncology* 2009; **27**(34): 5680-4.

4. Lamm DL, Blumenstein BA, Crissman JD, et al. Maintenance bacillus Calmette-Guerin immunotherapy for recurrent TA, T1 and carcinoma in situ transitional cell carcinoma of the bladder: a randomized Southwest Oncology Group Study. *The Journal of urology* 2000; **163**(4): 1124-9.

5. Holzbeierlein JM, Bixler BR, Buckley DI, et al. Diagnosis and Treatment of Non-Muscle Invasive Bladder Cancer: AUA/SUO Guideline: 2024 Amendment. *The Journal of urology* 2024; **211**(4): 533-8.

6. Bladder cancer: diagnosis and management of bladder cancer: © NICE (2015) Bladder cancer: diagnosis and management of bladder cancer. *BJU international* 2017; **120**(6): 755-65.

7. Steinberg G, Bahnson R, Brosman S, Middleton R, Wajsman Z, Wehle M. Efficacy and safety of valrubicin for the treatment of Bacillus Calmette-Guerin refractory carcinoma in situ of the bladder. The Valrubicin Study Group. *The Journal of urology* 2000; **163**(3): 761-7.

8. Balar AV, Kamat AM, Kulkarni GS, et al. Pembrolizumab monotherapy for the treatment of high-risk non-muscle-invasive bladder cancer unresponsive to BCG (KEYNOTE-057): an open-label, single-arm, multicentre, phase 2 study. *The Lancet Oncology* 2021; **22**(7): 919-30.

9. Khosravanian MJ, Mirzaei Y, Mer AH, et al. Nectin-4-directed antibody-drug conjugates (ADCs): Spotlight on preclinical and clinical evidence. *Life sciences* 2024; **352**: 122910.

10. Donat SM, Shabsigh A, Savage C, et al. Potential impact of postoperative early complications on the timing of adjuvant chemotherapy in patients undergoing radical cystectomy: a high-volume tertiary cancer center experience. *Eur Urol* 2009; **55**(1): 177-85.

11. Liberman D, Lughezzani G, Sun M, et al. Perioperative mortality is significantly greater in septuagenarian and octogenarian patients treated with radical cystectomy for urothelial carcinoma of the bladder. *Urology* 2011; **77**(3): 660-6.

12. Kim D, Kim JM, Kim JS, Kim S, Kim KH. Differential Expression and Clinicopathological Significance of HER2, Indoleamine 2,3-Dioxygenase and PD-L1 in Urothelial Carcinoma of the Bladder. *Journal of clinical medicine* 2020; **9**(5).

13. Hayashi T, Seiler R, Oo HZ, et al. Targeting HER2 with T-DM1, an Antibody Cytotoxic Drug Conjugate, is Effective in HER2 Over Expressing Bladder Cancer. *The Journal of urology* 2015; **194**(4): 1120-31.

14. Zhao J, Xu W, Zhang Z, et al. Prognostic role of HER2 expression in bladder cancer: a systematic review and meta-analysis. *International urology and nephrology* 2015; **47**(1): 87-94.

15. Sanguedolce F, Russo D, Mancini V, et al. Human Epidermal Growth Factor Receptor 2 in Non-Muscle Invasive Bladder Cancer: Issues in Assessment Methods and Its Role as Prognostic/Predictive Marker and Putative Therapeutic Target: A Comprehensive Review. *Urologia internationalis* 2019; **102**(3): 249-61.

16. Moustakas G, Kampantais S, Nikolaidou A, Vakalopoulos I, Tzioufa V, Dimitriadis G. HER-2 overexpression is a negative predictive factor for recurrence in patients with non-muscle-invasive bladder cancer on intravesical therapy. *The Journal of international medical research* 2020; **48**(1): 300060519895847.

17. Bongiovanni L, Arena V, Vecchio FM, Racioppi M, Bassi P, Pierconti F. HER-2 immunohistochemical expression as prognostic marker in high-grade T1 bladder cancer (T1G3). *Archivio italiano di urologia, andrologia : organo ufficiale [di] Societa italiana di ecografia urologica e nefrologica* 2013; **85**(2): 73-7.

18. Meghani K, Cooley LF, Choy B, et al. First-in-human Intravesical Delivery of Pembrolizumab Identifies Immune Activation in Bladder Cancer Unresponsive to Bacillus Calmette-Guérin. *Eur Urol* 2022; **82**(6): 602-10.

19. Robert J. Clinical pharmacokinetics of epirubicin. *Clinical pharmacokinetics* 1994; **26**(6): 428-38.

20. Maffezzini M, Campodonico F, Manuputty EE, et al. Systemic absorption and pharmacokinetics of single-dose early intravesical mitomycin C after transurethral resection of non-muscle-invasive bladder cancer. *Urology* 2013; **82**(2): 400-4.

1. **Appendix 1**
   1. **1973 and 2004 WHO Bladder Cancer Grading Systems**

| Papillary Tumor | |
| --- | --- |
| 1973 WHO Bladder Cancer Grading | |
| Urothelial Carcinoma Grade 1 (G1) | Well-differentiated |
| Urothelial Carcinoma Grade 2 (G2) | Moderately differentiated |
| Urothelial Carcinoma Grade 3 (G3) | Poorly differentiated |
| 2004 WHO Bladder Cancer Grading | |
| Papillary Urothelial Neoplasm of Low Malignant Potential (PUNLMP) | |
| Low-grade papillary urothelial carcinoma | |
| High-grade papillary urothelial carcinoma | |
|  | |

- 1. **2017 UICC TNM Staging**

| Stage | Criteria |
| --- | --- |
| T (Primary Tumor) |  |
| Tx | Primary tumor cannot be assessed |
| T0 | No evidence of primary tumor |
| Ta | Non-invasive papillary carcinoma |
| Tis | Carcinoma in situ (CIS, also known as flat carcinoma) |
| T1 | Tumor invades subepithelial connective tissue |
| T2 | Tumor invades muscularis |
| T2a | Tumor invades superficial muscularis (inner 1/2) |
| T2b | Tumor invades deep muscularis (outer 1/2) |
| T3 | Tumor invades bladder wall surrounding tissue |
| T3a | Tumor invasion found microscopically |
| T3b | Macroscopic tumor invasion into bladder wall surrounding tissue (extravesical mass) |
| T4 | Tumor invades any of the following organs or tissues, such as the prostate, seminal vesicles, uterus, vagina, pelvic wall, or abdominal wall |
| T4a | Tumor invades the prostate, seminal vesicles, uterus, or vagina |
| T4b | Tumor invades the pelvic wall or abdominal wall |
| N (Regional Lymph Nodes) |  |
| Nx | Regional lymph nodes cannot be assessed |
| N0 | No regional lymph node metastasis |
| N1 | Metastasis in a single lymph node in the true pelvis (hypogastric, obturator, external iliac, presacral) |
| N2 | Metastasis in multiple lymph nodes in the true pelvis (hypogastric, obturator, external iliac, presacral) |
| N3 | Metastasis in common iliac lymph nodes |
| M (Distant Metastasis) |  |
| Mx | Distant metastasis cannot be assessed |
| M0 | No distant metastasis |
| M1 | Distant metastasis |
|  |  |
|  |  |
|  |  |
|  |  |
|  |  |
|  |  |
|  |  |
|  |  |

- 1. **Non-muscle-invasive bladder cancer (NMIBC) risk classification criteria**

| Chinese Guidelines for the Diagnosis and Treatment of Bladder Cancer (2022) | |
| --- | --- |
| Low-risk NMIBC | Primary, single, TaG1 (low-grade urothelial carcinoma, PUNLMP), diameter ≤3cm, no CIS (Tis)  (Note: All the above conditions must be met simultaneously) |
| Intermediate-risk NMIBC | All NMIBC not included in the low-risk and high-risk classifications |
| High-risk NMIBC | Meets any of the following:  • G3 (or high-grade urothelial carcinoma)  • T1 stage tumor  • CIS (Tis)  • Meets all of the following simultaneously: multiple, recurrent, and diameter >3cmTaG1G2 (or low-grade urothelial carcinoma) |
| Extremely high-risk NMIBC | Meets any of the following:  • T1G3 (high-grade urothelial carcinoma) with concurrent bladder CIS (Tis)  • Multiple, large, recurrent T1G3 (high-grade urothelial carcinoma)  • T1G3 (high-grade urothelial carcinoma) with concurrent prostatic urethral CIS (Tis) or invasion;  • Urothelial carcinoma with adverse histopathological subtype  • Lymphatic, vascular invasion  • Failure of Bacillus Calmette-Guérin (BCG) bladder instillation |
|  |  |

1. **Appendix 2. New York Heart Association Functional Classification**

| The New York Heart Association (NYHA) functional classification of heart disease in 1928 | |
| --- | --- |
| Class | Description |
| I | Patients with heart disease but no limitation in activity. Ordinary activity does not cause fatigue, palpitations, dyspnea, or angina. |
| II | Slight limitation of physical activity. No symptoms at rest, but ordinary activity results in fatigue, palpitations, dyspnea, or angina. |
| III | Marked limitation of physical activity. Symptoms are provoked by less than ordinary activity. |
| IV | Unable to engage in any physical activity. Symptoms of heart failure occur at rest, which are aggravated by physical activity. |

1. **Appendix 3. ECOG Performance Status**

| ECOG Performance Status Scale (ECOG) | |
| --- | --- |
| Class | Description |
| 0 | Fully active, able to carry on all pre-disease activities without restriction. No evidence of disease. |
| 1 | Restricted in physically strenuous activities but ambulatory and able to carry out work of a light or sedentary nature, e.g., light house work, office work. |
| 2 | Ambulatory and capable of all self-care but unable to carry out any work activities up and about more than 50% of waking hours. |
| 3 | Limited self-care and confined to bed or chair more than 50% of waking hours. |
| 4 | Completely disabled and cannot carry on any self-care. Totally confined to bed or chair. |
| 5 | Dead |
|  |  |

1. **Appendix 4. Reference Formulas**
2. Body Surface Area: Body Surface Area = 0.0061 × height (cm) + 0.0124 × weight (Kg) - 0.0099
3. Cockcroft-Gault Formula Male: Creatinine Clearance Rate = (140 – age (years)) × weight (kg) / (72 × serum creatinine (mg/dL)) Female: Creatinine Clearance Rate = 0.85 × (140 – age (years)) × weight (kg) / (72 × serum creatinine (mg/dL))
4. **Appendix 5. Dose Escalation During Maintenance Infusion**

If a higher dose level is confirmed to be safe and tolerable, subjects at the lower dose level may switch to a higher dose if they meet all of the following criteria:

1. The subject has completed the DLT observation period.
2. The investigator assesses that it is in the best interest of the subject.
3. The subject has not experienced any grade 3 or higher treatment-related adverse events or other treatment-related toxicities that the investigator deems clinically significant.
4. The subject has not experienced persistent/recurrent NMIBC or disease progression.
5. Before dose escalation, the investigator's assessment and proposal are required, and confirmation of the number of donated drug vials must be obtained from RemeGen, Ltd.
